# Functions of block of proliferation 1 during anterior development in *Xenopus laevis*

**DOI:** 10.1371/journal.pone.0273507

**Published:** 2022-08-25

**Authors:** Corinna Gärtner, Annika Meßmer, Petra Dietmann, Michael Kühl, Susanne J. Kühl

**Affiliations:** 1 Institute of Biochemistry and Molecular Biology, Ulm University, Ulm, Germany; 2 International Graduate School in Molecular Medicine Ulm, Ulm, Germany; University of Colorado Boulder, UNITED STATES

## Abstract

Block of proliferation 1 (Bop1) is a nucleolar protein known to be necessary for the assembly of the 60S subunit of ribosomes. Here, we show a specific *bop1* expression in the developing anterior tissue of the South African clawed frog *Xenopus laevis*. Morpholino oligonucleotide-mediated knockdown approaches demonstrated that Bop1 is required for proper development of the cranial cartilage, brain, and the eyes. Furthermore, we show that *bop1* knockdown leads to impaired retinal lamination with disorganized cell layers. Expression of neural crest-, brain-, and eye-specific marker genes was disturbed. Apoptotic and proliferative processes, which are known to be affected during ribosomal biogenesis defects, are not hindered upon *bop1* knockdown. Because early *Xenopus* embryos contain a large store of maternal ribosomes, we considered if Bop1 might have a role independent of *de novo* ribosomal biogenesis. At early embryonic stages, *pax6* expression was strongly reduced in *bop1* morphants and synergy experiments indicate a common signaling pathway of the two molecules, Bop1 and Pax6. Our studies imply a novel function of Bop1 independent of ribosomal biogenesis.

## Introduction

Before cell division, cells need to grow so that daughter cells are similar in size to their parental cells. Cell growth occurs during the G1 phase of the cell cycle and requires a sufficient number of ribosomes. Ribosomes are involved in the cellular protein synthesis machinery that translates the genetic information from intermediate mRNA into a protein and are therefore crucial for cellular function [1]. Ribosomes are generated mainly in the nucleoli, and this process requires the synthesis of ribosomal proteins for the small and large ribosomal subunits and the transcription and processing of the 47S rRNA precursor (45S in *Xenopus*). The processing of the precursor is a complex step and requires numerous proteins to be organized into different pre-ribosomal multiprotein complexes. Defects in ribosome assembly or function can lead to a group of diseases called ribosomopathies. The best-known ribosomopathies are Diamond Blackfan anemia, Shwachman Diamond syndrome, and Treacher Collins syndrome [2,3].

Block of Proliferation 1 (Bop1) is one of the 200 factors known to be necessary for ribosomal synthesis [4–6]. Bop1 forms a protein complex with WD repeat domain 12 (WDR12) and Pescadillo homologue 1 (Pes1). This complex is called the PeBoW (Pes1-Bop1-WDR12) complex, and its integrity is required for rRNA processing and thus ribosomal biogenesis, as well as for cell proliferation [7,8]. Bound into the PeBoW complex, Bop1 is involved in the maturation of 28S and 5.8S rRNA and is therefore required for the assembly of the 60S subunit of the eukaryotic 80S ribosome [6]. Consistently, in mouse-derived cell lines dominant-negative mutants of Bop1 lead to cell cycle arrest in the G1 phase, resulting in impaired proliferation [9,10]. *bop1* knockdown also leads to drastically decreased protein synthesis in human cell lines [11].

*Xenopus laevis* oocytes can rely on a large store of 10^12^ maternal ribosomes, proteins, and mRNAs [12,13]. Therefore, their early development does not require the zygotic synthesis of new ribosomes, as shown in the anucleolate mutant. In homozygous anucleolate mutants, no rRNA precursors can be synthesized because of the lack of the rRNA gene cluster. Nevertheless, these embryos survive until the swimming tadpole stage [14]. During *Xenopus* embryogenesis, the nucleoli are reformed in the gastrula stage, which is accompanied by rRNA precursor synthesis [15] and ribosomal protein mRNA synthesis, both at low levels [16]. Significant ribosomal protein synthesis subsequently starts at stage 26 [16].

We previously analyzed the function of Pes1, one protein of the PeBoW complex, during early *Xenopus laevis* development, in particular during neural [17] and pronephric [18] development. We also analyzed the function of Peter Pan (Ppan), a Pes1-interacting protein, during *Xenopus* development [18,19]. We found a tissue-specific expression of these two genes, in e.g., the anterior neural plate, developing neural crest cells (NCCs), the eye and the pronephros. Loss-of-function studies indicated a crucial role of these proteins during early neural and pronephros development [17–19]. In neither neural nor pronephric tissues, the early phenotypes could be mimicked by interfering with 45S rRNA precursor processing, indicating a function of these proteins beyond their role in rRNA processing. In further studies, we were able to show that Ppan defines a tumor protein p53 (Tp53) independent of the nucleolar stress response pathway [20].

The above findings raised the interesting question of whether interfering with Bop1 function would also affect early *Xenopus* development. First evidence of a tissue-specific expression of *bop1* during early development in *Xenopus laevis* was provided by Neilson and colleagues [21]. To obtain further evidence, we therefore investigated the role of Bop1 during early *Xenopus* development. A detailed expression analysis of *bop1* revealed a specific expression during early anterior development, and loss-of-function studies revealed a function of Bop1 during neural development, in particular during eye and cranial cartilage development. This role of Bop1 involves the transcription factor Pax6 (Paired box 6), at least in part.

## Materials and methods

### Xenopus laevis

All procedures were approved by the German state administration Baden-Württemberg (Regierungspräsidium Tübingen) and performed according to the German animal use and care law. Embryos of *Xenopus laevis* were generated and cultured as described in previous protocols [22]. Embryos were cultivated in 0.1 x Modified Barth’s saline with HEPES buffer (MBSH) at 12.5 to 20°C, and staging was done according to Nieuwkoop and Faber [23]. Fixation was performed with MEMFA(T) (0.1 M MOPS (pH 7.4), 2mM EGTA, 1 mM MgSO_4_, 4% formaldehyde, (0.1% Tween20)).

### Synteny analysis and protein alignment

Synteny analysis and protein alignment of Bop1 were performed with the NCBI Gene Bank for *Homo sapiens* (NP_056016; Gene ID: 23246), *Danio rerio* (NP_001071203; Gene ID: 777627), and *Mus musculus* (AAB19223; Gene ID: 12181) and the Xenbase platform (xenbase.org) for *Xenopus laevis* (NP_001080358; Gene ID: XB-GENE-6254226 (bop1. L); NP_001079852; Gene ID: XB-GENE-6255479 (bop1. S)). For protein alignments, the software CLC Main Workbench 8 (Qiagen bioinformatics, 2017) was used.

### Whole mount *in situ* hybridization

Whole mount in situ hybridization (WMISH) was performed with a digoxygenin-labeled probe generated via *in vitro* transcription with T7, SP6, or T3 RNA polymerase (Roche) against the mRNA of different antisense probes in accordance with previous protocols [24,25]. We cloned the open reading frame of *Xenopus laevis bop1* into the pSC-B vector (Stratagene) with the cloning primers bop1_l 5’ GACAGGGAAAAACGTGTTTCT 3’ and bop1_r 5‘ TTATGTGAATAATCGGATTGTGG 3’. *In vitro* transcription with T3 RNA polymerases (Roche) resulted in digoxygenin-labelled antisense RNA probes. We used the following RNA anti-sense probes: *celf1* (*CUGBP Elav-like family member 1*) [26], *cryba1 (crystallin beta A1)* [26], *emx1* (*empty spiracles homeobox 1*) [27], *en2* (*engrailed homeobox 2*) [28], *egr2* (*early growth response 2*) [27], *foxd3* (*forkhead box D3*) [29], *otx2 (orthodenticle homeobox 2*) [30], *pax6* [31,32], *pou4f1* (*POU class 4 homeobox 1*) [33], *prox1* (*prospero homeobox 1*) [34], *rax (retina and anterior neural fold homeobox*) [35], *sox2* (*sex determining region Y-box 2*) (sequence ID: LC164013.1), *sox3* (sex *determining region Y-box 3*) [36], *vsx1* (*visual system homeobox 1*) [37], and *rho* (*rhodopsin*) [38]. For analyzing the expression of genes upon knockdown of *bop1* via WMISH, only the described tissues were evaluated.

### Morpholino oligonucleotide specificity and effectiveness test

Morpholino oligonucleotides were designed to the sequence of the *bop1* S homeologue. To show the specificity of the morpholino oligonucleotide (MO) binding, we cloned the MO binding sites and the binding sites as they appear on the *bop1* L homeologue in front of and in frame with the *green fluorescent protein* (*GFP)* gene by following an established protocol [17]. The cloning primers were as follows:

bop1_MO_bs_GFP_l, 5’ GATCCACCCGAGCCCTGAGTGAGACATGAAG 3’; bop1_MO_bs_GFP_r, 5’ AATTCTTCATGTCTCACTCAGGGCTCGGGTG 3’; bop1_MO2_bs_GFP_l, 5’ GATCCGAGACAGGGAAAAACGTGTTTCTACG 3’; bop1_MO2_bs_GFP_r, 5’ AATTCGTAGAAACACGTTTTTCCCTGTCTCG 3’; bop1_MO_bs_ChrL_GFP_l, 5’ GATCCCCCGAGTCCTGAGTGAAACATGAAG 3’; bop1_MO_bs_ChrL_GFP_r, 5’ AATTCTTCATGTTTCACTCAGGACTCGGGG 3’; bop1_MO2_bs_ChrL_GFP_l, 5’ GATCCGAGACAGGGAGAAACGTGTTTGTGCG 3’; bop1_MO2_bs_ChrL_GFP_r, 5’ AATTCGCACAAACACGTTTCTCCCTGTCTCG 3’; Δ5’UTRbop1_MO_bs_GFP_l, 5’ GATCCCCACTGTGGAATTCGCCCTTATGAAG 3’; and Δ5’UTRbop1_MO_bs_GFP_r, 5’ AATTCTTCATAAGGGCGAATTCCACAGTGGG 3’.

To test the MO binding specificity, we injected 1 ng of the respective *GFP* construct bilaterally together with 10 ng of *bop1* MO, *bop1* MO2, or Control MO into embryos at the two-cell stage and checked for GFP expression with an Olympus MVX10 fluorescence microscope.

### Morpholino oligonucleotides, cloning, injection mRNAs, and microinjections

We designed two *bop1* MOs that bound to the following sequences: *bop1* MO, 5’-CCCGAGCCCUGAGUGAGACAUGAA-3’; and *bop1* MO2, 5’-GAGACAGGGAAAAACGUGUUUCUAC-3’. *pax6* MO and *tp53* MO were used as previously described [39,40]. All gene-specific and a standard Control MO were obtained from Gene Tools (Philomath, OR, USA). MOs were diluted in water treated with diethyl pyrocarbonate (DEPC) and injected into one of the two animal-dorsal blastomeres of eight-cell embryos to specifically target the anterior tissue. As an injection control, 0.5 ng of *GFP* RNA was co-injected, and the fluorescence was assessed with a fluorescence microscope (Olympus MVX10, U-RFL-T, Japan). The uninjected side served as an internal control and Control MO injections, as an injection control. If not indicated differently, the following amounts of MO were injected: 15 ng *bop1* MO, 15 ng *bop1* MO2, 15 ng Control MO, 15 or 30 ng *pax6* MO, and 2.5 or 5 ng *tp53* MO. For rescue experiments *Xenopus* Δ*5’UTR-bop1* RNA was cloned into the pCS2+ vector (Rupp and Weintraub) using *Xho*I and *Xba*I for restriction and the following primers: Δ5’UTRbop1_l: 5’ ATGAAGAGAGGGAGCCAAGGGGAG 3’ and Δ5’UTRbop1_r: 5’ TTATGTGAATAATCGGATTGTGG 3’. Generation of injection mRNAs was accomplished by *in vitro* transcription using T3, T7, or SP6 polymerase. Rescue experiments of Bop1 were performed by injecting *bop1* MO along with 0.5 ng of *Xenopus* Δ*5’UTR-bop1* RNA, which is not targeted by the *bop1* MO and *bop1* MO2, into one animal-dorsal blastomere (Δ*5’UTR-bop1* RNA is not targeted by the *bop1* MO and *bop1* MO2 because of the altered sequence as shown in S2B Fig). *bop1* RNA for gain of Bop1 function experiments *bop1* RNA was cloned into the pCS2+ vector using *Xho*I and *Xba*I for restriction and the following primers: bop1_l: 5’ GACAGGGAAAAACGTGTTTCT 3’ and bop1_r: 5’ TTATGTGAATAATCGGATTGTGG 3’. Gain-of-function experiments were implemented with injections of *bop1* RNA ranging from 0.5 to 1 ng. For synergy experiments, 5 ng *pax6* MO and 5 ng of *bop1* MO were injected unilaterally alone or in combination. Co-injections of *bop1* MO and *foxd3* RNA [29] were carried out with 50, 250, or 750 pg *foxd3* RNA. Co-injection of *bop1* MO and *c-myc* (*MYC proto-oncogene*) RNA [19] were carried out with 0.5 ng *c-myc* RNA. Co-injections of *bop1* MO together with *pax6* RNA were performed with 0.2 ng, 0.5 ng, or 1 ng *pax6* RNA. *GFP* RNA was used to adjust RNA levels for all experiments in which RNA was injected and Control MO was used to adjust MO levels for all experiments in which MOs were injected.

### Quantitative real-time PCR

Embryos were bilaterally injected with 20 ng Bop1 MO or 20 ng Control MO at two-cell stage. At stage 13, total RNA was isolated from whole embryos using peqGOLD RNAPure Kit (PEQLAB) according to the manufacturers’ protocols. cDNA synthesis was performed using Superscript II reverse transcriptase and random primers (Invitrogen). Primers for qPCR were designed using the online tool NCBI primer blast. The following primers were used:

slc35b1_qPCR_l: 5’ CGCATTTCCAAACAGGCTCC 3’ [41]

slc35b1_qPCR_r: 5’ CAAGAAGTCCCAGAGCTCGC 3’ [41]

odc_qPCR_l: 5’ TGCACATGTCAAGCCAGTTC 3’ [42]

odc_qPCR_r: 5’ GCCCATCACACGTTGGTC 3’ [42]

glyceradehyde-3-phosphate dehydrogenase (gapdh)_qPCR_l: 5’ GCCGTGTATGTGGTGGAATCT 3’ [19]

gapdh_qPCR_r: 5’ AAGTTGTCGTTGATGACCTTTGC 3’ [19]

sox2_qPCR_l: 5’ AACTCTGCGTCCAACAACCA 3’

sox2_qPCR_r: 5’ TGTGCATCTTGGGGTTCTCC 3’

sox3_qPCR_l: 5’ GGATCAGGATCGGGTGAAGC 3’

sox3_qPCR_r: 5’ GCTGATCTCCGAGTTGTGCA 3’

For quantitative real-time PCR, the QuantiTect SYBR Green PCR Kit (Qiagen) and a BioRad CFX Connect Real Time System was used. *gapdh*, *slc35b1*, and *odc* served as housekeeping genes. Each experiment was measured in triplicates and qPCR products were verified via gel electrophoresis. Relative gene expression was calculated using the ΔΔCT method and the three housekeeping genes as previously described [43].

### Knockout using the CRISPR/Cas9 system

For gene editing experiments using the clustered regularly interspaced short palindromic repeats (CRISPR)/ CRISPR-associated protein 9 (Cas9) system, *bop1 gRNA #1* was designed using CRISPRscan (crisprscan.org) [44] targeting exon 8 of the *bop1* S and *bop1* L homeologue with the following CRISPR site: 5’ TGTACCTGTGCCCGCGCCAG 3’. The gRNA template was synthesized using the oligo extension reaction [45]. Transcription of gRNA was performed using the MEGAshortscript T7 Transcription kit (Invitrogen) and the miRNAeasy Micro kit (Qiagen) for purification. Cas9 protein (PNA Bio) and gRNA were assembled to the ribonucleoprotein complex by 5 min incubation at 37°C. 1 ng Cas9 protein was injected together with 300 pg gRNA. For phenotype analysis, embryos were injected at two-cell stage. Cas9 protein alone and injection of 0.5 ng *GFP* RNA into the second blastomere served as injection control. For sequencing experiments, embryos were injected at one-cell stage and lysed (50 mM Tris [pH 8.8], 1 mM EDTA, 0.5% Tween 20, 200 μg/ml proteinase K) at stage 43. DNA was extracted as previously described [45]. DNA pools of 10 control embryos or 10 *bop1 gRNA #1* injected embryos were combined. The following primer were used for confirming gene editing via direct sequencing:

bop1 gRNA #1 6L-l: 5’ GTATGTTCCATCTCACTTCCTGC 3’

bop1 gRNA #1 6L-r: 5’ GTACCAGTGCAGGGAAACAAT 3’

bop1 gRNA #1 6S-l: 5’ TCTCTTCCCCTGTTGGCTCCT 3’

bop1 gRNA #1 6S-r: 5’ AAGACATGTAGCGGCAGTGTAA 3’.

### Alcian blue staining

Cranial cartilage staining was performed according to Gessert et al. [17]. *bop1* MO-injected embryos were fixed at stage 45 in 1x MEMFA for 1 hour and stained overnight at room temperature in a staining solution containing 1% Alcian blue and 0.5% acetic acid diluted in water. To wash the embryos, we used 80% EtOH / 20% acetic acid, changing the solution several times. Bleaching was performed with 30% H_2_O_2_. Manual isolation of cartilage was reached with fine tweezers.

### Imaging

*Xenopus* embryos were imaged with an Olympus MVX10 (fluorescence) or Olympus SZX12 microscope and an Olympus UC50 camera. Sections were imaged with an Olympus BX60 microscope and an Olympus DP70 camera. Images were processed with ImageJ64, Affinity Designer 1.10.4, Adobe Photoshop CS6, and Adobe Illustrator CS5.

### Quantitative tissue measurements

The head width (distance between midline and the lateral edge of the embryo), the area of the eye, and the coloboma (apex angle in degree) were measured with the software ImageJ64 (Wayne Rasband, NIH). For the analysis of brain size, embryos were fixed at stage 42, the brain was isolated, and pictures were taken. ImageJ was used to measure the brain area. For the analysis of the width of *sox3* expression, embryos were fixed at stage 13 and stained with a *sox3* antisense probe. After imaging, the width of *sox3* expression was measured with ImageJ. To analyze the area of *sox3* and *pax6* expression, *bop1* MO and Control MO-injected embryos were photographed after WMISH. By using ImageJ, the area of expression was selected and measured. The intensity of GFP expression was analyzed using ImageJ. Here, the mean of the grey values was analyzed at indicated areas and normalized to the control group.

### Histology sections

A vibrating microtome (Vibratome 1500 Classic, The Vibratome Company) was used to slice 25 μm-thick sections of the *Xenopus* embryos. Before sectioning, the embryos were embedded in blocks containing gelatin and glutaraldehyde.

### Phospho histone 3 staining

For analysis of cell proliferation, phospho histone 3 (pH3) staining was performed in unilaterally injected embryos at stage 23 that were fixed with MEMFAT for 2 hours at room temperature. First, embryos were washed in 1x PBS for 5 x 10 minutes and then blocked for 1 hour in 1x PBS / 10% Horse Serum before being treated with a rabbit-anti-pH3 antibody (1:100; Millipore, Temecula, CA, USA), diluted in 1x PBS / 10% Horse Serum and incubated overnight at 4°C. The next day, embryos were washed in a solution of 1x PBS / 0.1% Tween-20 for 2 hours, whereby the medium was changed 4 times. For blocking, embryos were incubated in 1x PBST / 20% Horse Serum for 1 hour. Incubation with the secondary antibody was performed for 5 to 6 hours with 1x PBST / 20% Horse Serum / anti-rabbit-IgG-AP (1:1000; Sigma-Aldrich, Munich, Germany). Overnight, embryos were kept in 1x PBS / 0.1% Tween at 4°C. On the last day, embryos were transferred into a 24-well plate after several washing steps with 1x PBS / 0.1% Tween and then incubated with AP buffer for 10 minutes. Staining was performed with 500 μl of BM purple, and fixation, with MEMFAT. 30% H_2_O_2_ was used to bleach the. Then, pH3-positive cells were counted on both sides of the eye area.

### Terminal deoxynucleotidyl transferase dUTP nick end labeling assay

Cell apoptosis was detected via terminal deoxynucleotidyl transferase dUTP nick end labeling (TUNEL) staining. TUNEL staining was performed at stage 23 according to established protocols [17,27,46]. After fixation, embryos were bleached in 30% H_2_O_2_. TUNEL-positive cells were counted on both sides of the embryo.

### Statistics

Data were analyzed with the software GraphPad Prism 9 (San Diego, CA, USA). P values were calculated with a one-tailed, non-parametric Mann-Whitney rank sum test. For real-time qPCR analysis a paired t-test was performed upon checking for normal distribution using a Shapiro-Wilk test. Error bars represent standard error of the means (SEM). Statistical significance was indicated as follows: *, p ≤ 0.05; **, p ≤ 0.01; ***, p ≤ 0.001; and ****, p ≤ 0.0001.

## Results

### Genomic analysis of *bop1*

To investigate the genomic region of *bop1*, we performed an *in silico* synteny analysis in which we compared the genomes of *Homo sapiens*, *Mus musculus*, *Xenopus laevis* (both homeologues), and *Danio rerio* (S1A Fig). This approach showed that the neighboring genes of *bop1* are highly conserved across species, although some deletions and inversions were observed upstream and downstream of *bop1*. A protein alignment showed that the proteins are similar between the various species, especially at the BOP1 N-terminal (NT) domain and the WD40 repeat (WD 40) domain (S1B–S1D Fig). Taken together, these data suggest a strong conservation of Bop1 across species.

### Spatiotemporal expression of *bop1* during early *Xenopus* embryogenesis

**Fig 1:**
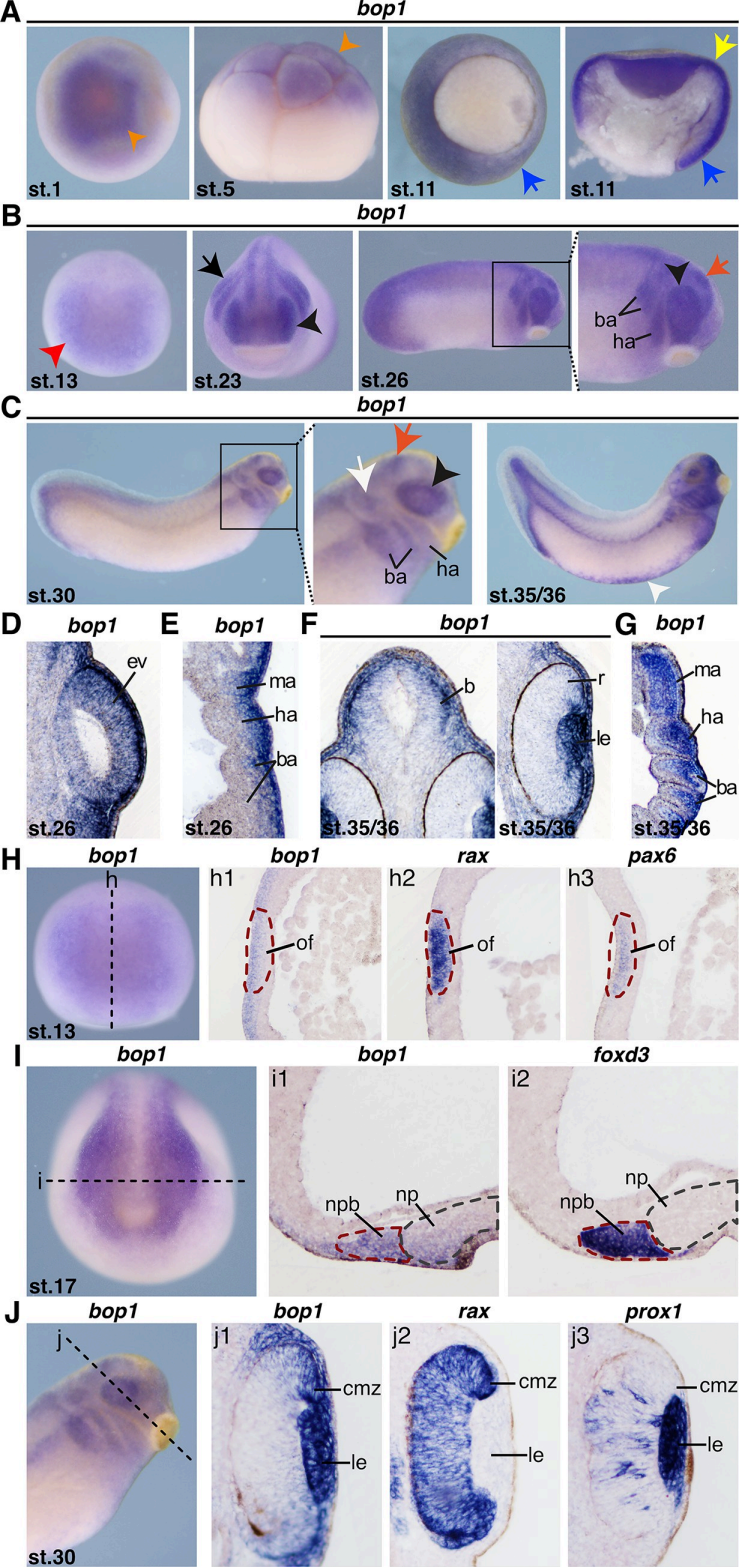
*bop1* expression in *Xenopus laevis* embryos. **A** *bop1* expression visualized by whole mount *in situ* hybridization in embryos at the indicated stages. Animal (st. 1), lateral (st. 5), and vegetal (st. 11) views and a sagittal section (st. 11) are shown. At stage 1 and stage 5, *bop1* was expressed at the animal pole (orange arrowhead). During gastrulation (st. 11), *bop1* expression occurred in the mesoderm surrounding the blastoporus (yellow and blue arrow). **B, C** Anterior (st. 13 and 23) and lateral (st. 26, st. 30, and st. 35/36) views are shown. At stage 13, *bop1* expression was found in the anterior neural plate (red arrowhead). Stage 23 and stage 26 embryos showed expression in the developing eye (black arrowhead), the migrating neural crest cells (NCCs) (black arrow), and the brain (red arrow). At tailbud stages, embryos expressed *bop1* in the developing eye (black arrowhead), the brain (red arrow), the branchial (ba) and hyoid arches (ha), the otocyst (white arrow), the blood islands (white arrowhead), and the tail bud. **D** *bop1* expression was shown in the developing eye (transversal section, orientation: Dorsal (upper part) to ventral (lower part)). **E, G** *bop1* expression was shown in the branchial, hyoid, and mandibular arches (ma) (horizontal sections, orientation: Anterior (upper part) to posterior (lower part)). **F** Embryos expressed *bop1* in the brain (b) and in the lens (le) (transversal section, orientation: Dorsal (upper part) to ventral (lower part)). **H** Expression of *bop1* and the two eye-specific marker genes *rax* and *pax6* at stage 13. The black dotted line indicates the level of sagittal sections in h1-h3 (section orientation is dorsal (upper part) to ventral (lower part)). *bop1* expression was detected in the optic field (of) (red dotted circle), as was *rax* and *pax6* expression. **I** Expression of *bop1* and the NCC marker gene *foxd3* at stage 17. The black dotted line in the left-hand panel represents the level of horizontal sections in i1 and i2 (section orientation is posterior (upper part) to anterior (lower part)). *bop1* transcripts were located in the neural plate border positive for *foxd3* (nbp) (red dotted line). The grey dotted line in the middle and right-hand panels represents the neural plate (np). **J** Expression of *bop1* and the two eye-specific marker genes *rax* and *prox1* at stage 30. The black dotted line indicates the level of transversal sections shown in j1-j3 (section orientation is dorsal (upper part) to ventral (lower part)). *bop1* was expressed in the lens and the ciliary marginal zone (cmz), where retinal progenitor cells are located. Abbreviations: b, brain; ba, branchial arches; cmz, ciliary marginal zone; ev, eye vesicle; ha, hyoid arches; le, lens; ma, mandibular arches; NCC, neural crest cell; np, neural plate; nbp, neural plate border; of, optic field; r, retina; st., stage; WMISH, whole mount *in situ* hybridization.

To investigate the expression pattern of *bop1* in *Xenopus laevis* embryos, we used WMISH approaches with a *bop1-*specific antisense RNA probe (Fig 1). *bop1* is expressed at the animal pole in very early stages of development (Fig 1A). During gastrulation, *bop1* expression was detected in the ectoderm and the invaginating mesoderm surrounding the blastoporus (Fig 1A). At stage 13, *bop1* expression was found in the anterior neural plate and in the optic field, where *rax* and *pax6* are expressed as well (Fig 1B and 1H). At stage 17, *bop1* expression was located in the neural plate border, where NCCs are induced as shown by comparison with *foxd3* expression (Fig 1I). Stage 23 embryos showed *bop1* transcripts in the eye anlage and the migrating NCCs (Fig 1B). In later stages, *bop1* mRNA was visualized in the developing eye, the brain, the NCCs of the mandibular, hyoid and branchial arches, the blood islands, the otic vesicle, and the tailbud (Fig 1B and 1C). Vibratome sections confirmed these findings (Fig 1D–1G). At stage 30 and 35/36, expression of *bop1* was located in the ciliary marginal zone and the lens, as confirmed by expression of *rax* and *prox1*, respectively (Fig 1F and 1J).

### *bop1* knockdown leads to defects during NCC development

Since *bop1* was shown to be specifically expressed in the early anterior tissue (Fig 1), we analyzed the function of Bop1 in *Xenopus* embryos by loss-of-function experiments with a potent antisense MO-based approach. Since Session and colleagues have shown by RNA sequencing data that both homeologues (S and L) are expressed in the here addressed tissues [47], two different *bop1* MOs against *Xenopus bop1* mRNA were used; *bop1* MO and *bop1* MO2, both of which showed a high binding specificity to their binding sites on both *bop1* homeologues S and L (S2A–S2D Fig).

**Fig 2:**
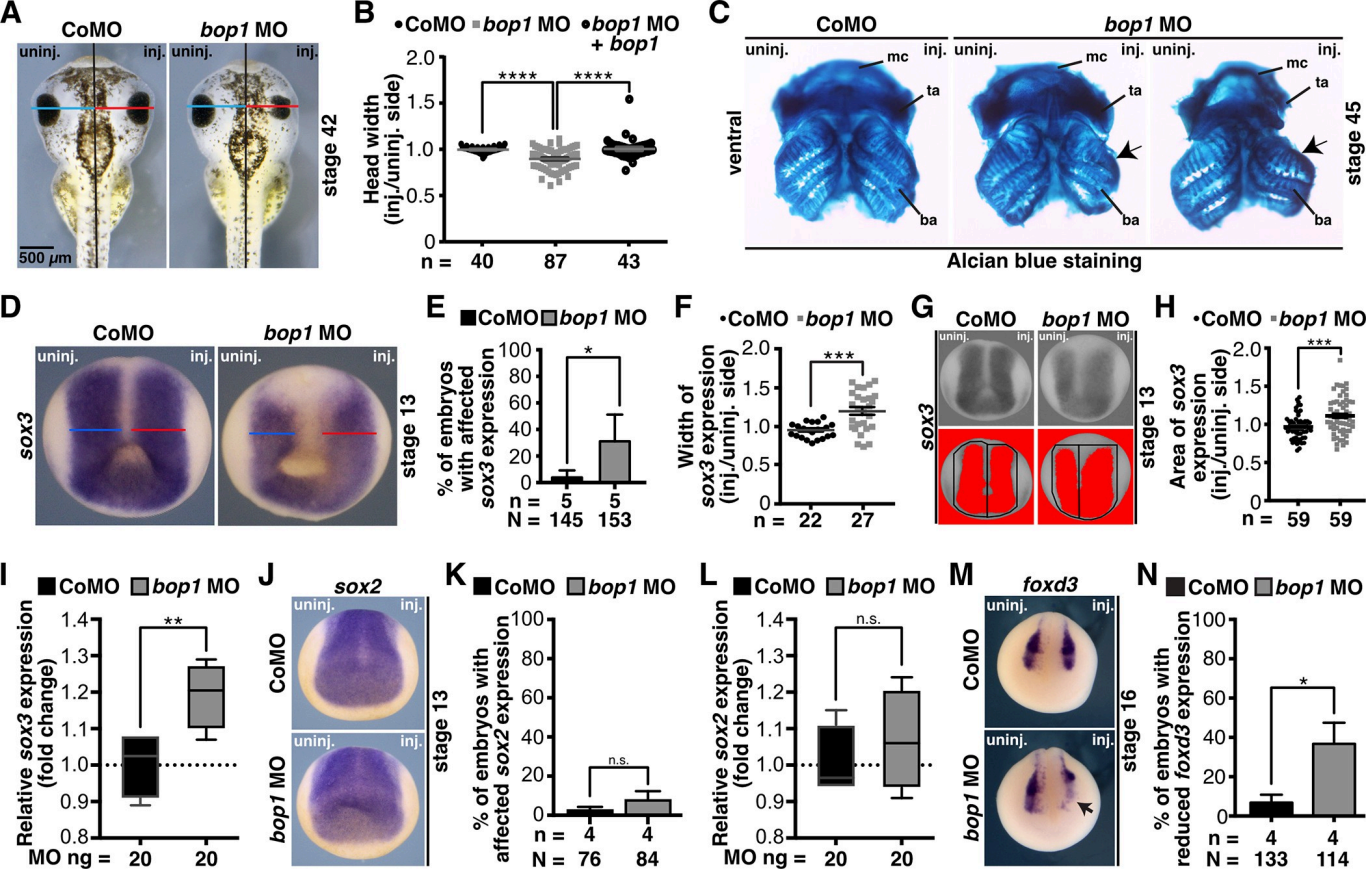
*bop1* knockdown leads to a cranial cartilage phenotype. **A** The head width on the injected side (red line) of embryos at stage 42 injected with *bop1* MO and Control MO was compared with the head width on the uninjected side (blue line). The head was significantly smaller upon *bop1* knockdown. **B** Statistical evaluation of data given in A. The head phenotype was rescued by co-injecting *bop1* MO with 0.5 ng of Δ*5’UTR-bop1* RNA. **C** Ventral view of Alcian blue-stained cranial cartilages of stage 45 embryos showed a reduced cartilage upon *bop1* knockdown. The middle cartilage depicts a mild phenotype and the right one, a severe phenotype. Branchial arches (ba), Meckel´s cartilage (mc), tectum anterius (ta) were severely affected (black arrows). **D** *sox3* served as pan-neural marker gene at stage 13 and was analyzed upon *bop1* and Control MO injection. Anterior view of embryos is given. The width of *sox3* expression on the injected side (red line) of *bop1* MO- and Control MO-injected embryos at stage 13 was compared with the width on the uninjected side (blue line). **E** *sox3* expression significantly increased in *Xenopus* embryos upon *bop1* MO injection. **F** Statistical evaluation of data given in D showing an increased width of *sox3* expression in *bop1* morphants. **G** *sox3* expression was analyzed in stage 13 *bop1* morphants and control embryos (anterior view is shown). Expression domain was photographed and area was measured via ImageJ. Black boxes indicate area where *sox3* expression (red area) was measured. Injected side was compared to uninjected side. **H** *sox3* expression is significantly increased in *bop1* morphants. Control MO injection did not alter *sox3* expression. **I** Relative *sox3* gene expression was analyzed using real-time qPCR in *bop1* MO and Control MO bilaterally injected embryos at stage 13. *sox3* expression was normalized to *gapdh*, *odc*, and *slc35b1* and was significantly increased upon *bop1* knockdown compared to control MO injection. **J** Anterior view of embryos is given. Expression of *sox2* was analyzed upon *bop1* MO and Control MO injection at stage 13. Neither *bop1* MO nor Control MO injection affected *sox2* expression. **K** Statistical analysis of data shown in J. **L** Relative *sox2* gene expression was analyzed using real-time qPCR in *bop1* MO and Control MO bilaterally injected embryos at stage 13. *sox2* expression was normalized to *gapdh*, *odc*, and *slc35b1* and was not affected upon *bop1* knockdown. **M** The marker gene *foxd3* in anterior neural crest cells in stage 17 embryos injected with 20 ng of *bop1* MO or Control MO (black arrow indicates location of altered gene expression). **N** Around 40% of *bop1* MO-injected embryos showed a reduced expression of *foxd3* upon *bop1* MO injection, but Control MO injection did not show any effect on *foxd3* expression. Abbreviations: ba, branchial arches; *bop1* MO, block of proliferation 1 morpholino oligonucleotide; CoMO, Control MO; inj., injected side; mc, Meckel´s cartilage; MO, morpholino oligonucleotide; n, number of independent experiments; N, number of injected and analyzed embryos; n.s., non-significant; ta, tectum anterius; uninj., uninjected side. *bop1* is the Δ*5’UTR-bop1* RNA used for rescues. Error bars indicate standard error of the means; Whiskers indicate in I and L minimum and maximum. *, p ≤ 0.05; **, p ≤ 0.01; ***, p ≤ 0.001; ****, p ≤ 0.0001.

To target anterior (neural) tissue, we injected *bop1* MO unilaterally into one animal-dorsal blastomere of *Xenopus* embryos at eight-cell stage [48]. The uninjected side served as an internal control, and injection of a Control MO, as an injection control. To examine the phenotype upon loss of Bop1 function, embryos were analyzed at stage 42. Head width measurements revealed a significant decrease of the head width in *bop1* MO-injected embryos; Control MO-injected embryos showed normally developed heads (Fig 2A and 2B). Injection of *bop1* MO2 led to an identical phenotype in *Xenopus* embryos (S3A and S3B Fig). For rescue experiments, we generated a *Xenopus bop1* RNA (Δ*5’UTR-bop1* RNA) that is not targeted by *bop1* MO and *bop1* MO2 (S2B and S2C Fig). For the sake of simplicity, *Xenopus* Δ*5’UTR-bop1* RNA is referred to as *bop1* RNA in the figures. Co-injecting 15 ng *bop1* MO or 15 ng *bop1* MO2 together with 0.5 ng *Xenopus* Δ*5’UTR-bop1* RNA led to a rescue of the NCC-derived phenotype and thus demonstrated the specificity of the *bop1* MO-induced phenotype (Figs 2B and S3).

We also performed *bop1* gain-of-function experiments. Overexpressing *bop1* in *Xenopus* embryos did not result in a smaller head or any other phenotype of the anterior tissue (S4A and S4B Fig).

Since *bop1* expression was found in the NCCs (Fig 1) and NCCs migrate into cranial cartilage structures as one derivative [49], we investigated a potential role of Bop1 during NCC development. Hence, we analyzed cranial cartilage development by Alcian blue staining to detect cranial cartilage structures, at stage 45. In line with the finding of smaller head widths, *bop1* morphants showed a smaller cranial cartilage upon *bop1* knockdown (Fig 2C). Here, especially the Meckel´s cartilage and the tectum anterius were affected.

To analyze the molecular basis of the NCC-derived cranial cartilage phenotype, we first analyzed the expression of the pan-neural marker gene *sox3* at stage13. Upon *bop1* knockdown, *sox3* expression was significantly increased (Fig 2E). The *sox3* expression domain was found to be significantly broader upon Bop1 deficiency, as shown by analysis of the width as well as area of *sox3* expression (Fig 2D and 2F–2H). An increase in relative *sox3* expression was confirmed by real-time qPCR analysis (Fig 2I). Another pan-neural marker gene, *sox2*, was unaffected upon *bop1* knockdown, which was analyzed by WMISH and real-time qPCR approaches (Fig 2J–2L). Next, we examined the expression of the NCC-specific marker gene *foxd3* at stage 16, when NCCs are induced in the neural plate border. *foxd3* expression was significantly downregulated upon *bop1* MO injection. Control MO-injected embryos showed normally expressed *foxd3* (Fig 2M and 2N).

To investigate whether the NCC phenotype solely originates from disturbed *foxd3* expression, we performed rescue experiments by co-injecting *bop1* MO and *foxd3* RNA. The cranial cartilage phenotype was not rescued by co-injecting *foxd3* RNA (S4C–S4E Fig).

In conclusion, in *Xenopus laevis* loss of Bop1 function leads to smaller heads and cranial cartilage as well as defects in NCC induction.

### *bop1* knockdown affects proper brain development

**Fig 3:**
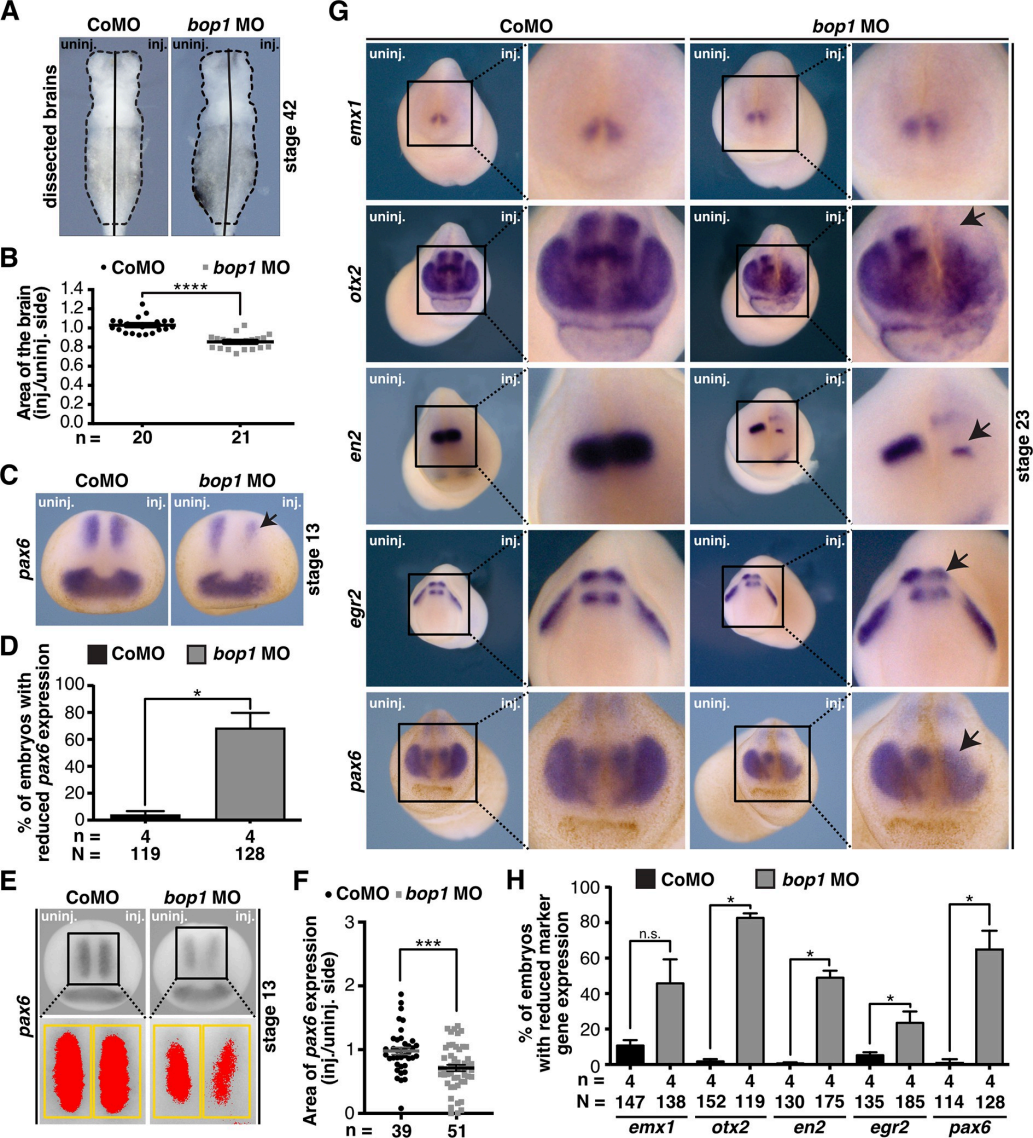
Bop1 is necessary for brain development in *Xenopus laevis*. **A** Sides of embryo brains injected with *bop1* MO and Control MO and uninjected sides were compared (ventral view). **B** Statistical evaluation showed that *bop1* knockdown led to a significantly smaller area of the brain. **C** *Xenopus* embryos were injected with either 20 ng *bop1* MO or Control MO, and *pax6* expression in the anterior neural tube was investigated at stage 13 (black arrow indicates location of reduced gene expression). *pax6* expression was decreased upon *bop1* knockdown. **D** Statistical evaluation of data given in C. **E** Area of *pax6* expression was investigated at stage 13 embryos via ImageJ. Expression domain was photographed and injected side was compared to uninjected side. Yellow boxes indicate area in which *pax6* expression (red area) was measured. **F** Statistical analysis confirmed reduction of *pax6* expression upon *bop1* knockdown. **G** Brain-specific marker genes were analyzed in stage 23 embryos. Embryos were injected with 20 ng *bop1* MO or Control MO. Black arrows indicate location of reduced gene expression. **H** As shown by statistical evaluation, upon *bop1* knockdown expression of *otx2*, *en2*, *egr2*, and *pax6* was significantly decreased. Control MO-injected embryos showed normal gene expression. Abbreviations: *bop1* MO, block of proliferation 1 morpholino oligonucleotide; CoMO, Control MO; inj., injected side; n, number of independent experiments; N, number of injected and analyzed embryos; n.s., non-significant; uninj., uninjected side. Error bars indicate standard error of the means; *, p ≤ 0.05; ***, p ≤ 0.001; ****, p ≤ 0.0001.

Since *bop1* was also found to be specifically expressed in the developing brain, we investigated brain development of *Xenopus* upon *bop1* knockdown (Fig 1). Therefore, we dissected brains of *bop1* MO- or Control MO-injected embryos at stage 42 and measured the area of the brain. Indeed, brains were significantly smaller on the *bop1* MO-injected side, whereas Control MO injection had no effect on brain development (Fig 3A and 3B).

The molecular basis of the brain phenotype was analyzed by WMISH and the well-known brain-specific marker genes *pax6* (forebrain and posterior neural tube), *emx1* (forebrain), *otx2* (fore- and midbrain), *en2* (mid-/hindbrain boundary, isthmus), and *egr2* (hindbrain). At stage 13, *pax6* expression was reduced in the anterior neural tube upon *bop1* knockdown (Fig 3S and 3D). A quantitative analysis confirmed the decrease of *pax6* expression (Fig 3E and 3F). At stage 23, the expression of the analyzed marker genes, *otx2*, *en2*, *egr2*, and *pax6* was reduced in *bop1* MO-injected embryos, whereas Control MO injection did not affect expression of analyzed genes (Fig 3G and 3H).

Taken together, Bop1 function was shown to be required for brain development in *Xenopus laevis* embryos.

### Bop1 is required for proper *Xenopus laevis* eye development

**Fig 4:**
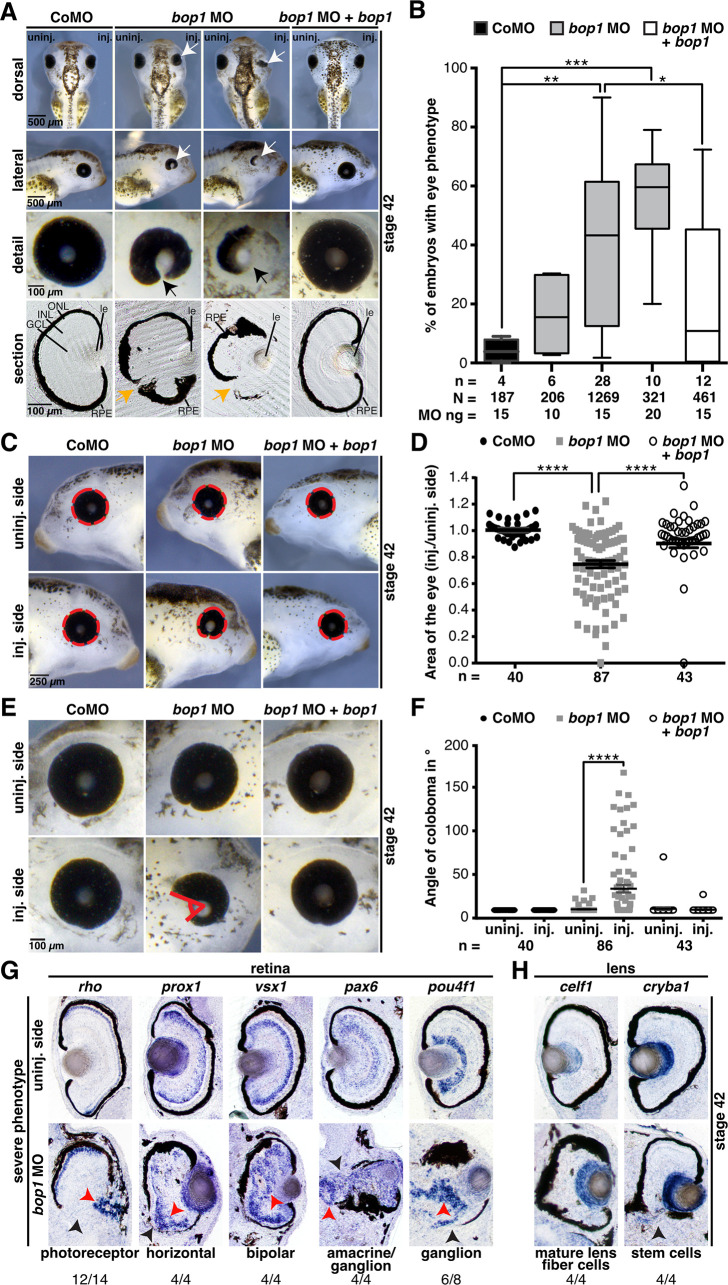
*bop1* knockdown impairs proper eye development. **A** Sides injected with *bop1* MO and Control MO were compared with uninjected sides of embryos. Knockdown of *bop1* led to a severe eye phenotype with underdeveloped or malformed eyes (white arrows). A detailed view of the deformed eyes is depicted (black arrows). Vibratome sections showed a deformed retinal pigmented epithelium (RPE) (orange arrows). Control MO-injected embryos showed no eye phenotype. **B** Statistical evaluation of data given in A. Embryos with Bop1 suppression developed a significantly smaller eye than Control MO-injected embryos. The phenotype increased in a dose-dependent manner and was rescued by co-injecting 0.5 ng of Δ*5’UTR-bop1* RNA. **C** The area of the eye was measured (red dotted circles). Control MO- and *bop1* MO-injected sides were compared with uninjected sides. **D** *bop1* knockdown led to significantly smaller eyes. The size of the eye was rescued by co-injecting Δ*5’UTR-bop1* RNA. **E** Measurement of the angle of eye fissures (indicated by red angle). **F** Statistical analysis of the angle of eye fissures showed that *bop1* MO-injected embryos developed colobomas. The coloboma phenotype was rescued by co-injecting *bop1* MO with Δ*5’UTR-bop1* RNA. **G** Vibratome sections of *bop1* MO-injected embryos showing a severe eye phenotype. Red arrows point to disrupted cell layers of indicated cell types, and black arrows point to disrupted RPE. Specific marker genes for cell layers were used, as described in the main text. Most of the cell types were displaced and disorganized. **H** Lens-specific cells were unimpaired upon suppression of Bop1. Vibratome sections of embryos showing a severe eye phenotype. The lens-specific marker genes *celf1* and *cryba1* were used. Some lens fiber cells and stem cells were mildly displaced, but they showed no reduction in expression. Numbers below the columns indicate the number of embryos showing the depicted phenotypes per number of embryos analyzed. Abbreviations: *bop1* MO, block of proliferation 1 morpholino oligonucleotide; CoMO, Control MO; GCL, ganglion cell layer; inj., injected side; INL, inner nuclear cell layer; le, lens; n, number of independent experiments; N, number of injected and analyzed embryos; ONL, outer nuclear cell layer; RPE, retinal pigmented epithelium; uninj., uninjected side. *bop1* is the Δ*5’UTR-bop1* RNA used for rescues. Error bars indicate standard error of the means; Whiskers in B indicate minimum and maximum. *, p ≤ 0.05; **, p ≤ 0.01; ***, p ≤ 0.001; ****, p ≤ 0.0001.

As shown in Fig 1, *bop1* expression was found in the developing eye of *Xenopus laevis* embryos. Hence, we analyzed the eyes of *bop1* morphants at stage 42, and found eyes to be smaller and deformed. The uninjected side and Control MO-injected embryos showed no phenotype (Fig 4A). This phenotype was induced in a MO dose-dependent manner (Fig 4B). Vibratome sections revealed an impaired retinal pigmented epithelium (RPE) upon Bop1 deficiency (Fig 4A). By co-injecting Δ*5’UTR-bop1* RNA, the eye defects were rescued (Fig 4A and 4B).

As a quantitative evaluation, we measured the eye area after *bop1* and Control MO injections and detected a significant decrease of the eye area in *bop1* MO-injected embryos. The phenotype was rescued by co-injecting 0.5 ng Δ*5’UTR-bop1* RNA (Fig 4C and 4D). In addition, we measured the angle of the eye fissure and observed a severe coloboma phenotype after *bop1* MO injection but a normal phenotype after Control MO injection. Again, co-injection of Δ*5’UTR-bop1* RNA resulted in a rescue of the coloboma phenotype (Fig 4E and 4F). Injection of *bop1* MO2 led to identical eye phenotypes in *Xenopus* embryos, which were also rescued by co-injecting *Δ5’UTR-bop1 RNA* (S3C–S3H Fig).

Since sections of the eye showed an impaired RPE in *bop1* morphants, we performed WMISH experiments with well-known retina cell type-specific marker genes [27]. In detail, we used *rho* for photoreceptor cells, *prox1* for horizontal cells, *vsx1* for bipolar cells, *pax6* for amacrine and ganglion cells, and *pou4f1* for ganglion cells. Vibratome sections revealed a massive disruption of the retinal layering upon *bop1* knockdown but normal development in the uninjected retinas. In particular, photoreceptor cells formed rosette-like structures (Fig 4G).

*bop1* is specifically expressed in the developing lens, hence we investigated lens development upon *bop1* knockdown by WMISH with the specific marker genes *celf1* for lens fiber cells and *cryba1* for lens stem cells. Neither *celf1* nor *cryba1* expression was downregulated in Bop1-depleted lenses in comparison with the uninjected lenses (Fig 4H).

**Fig 5:**
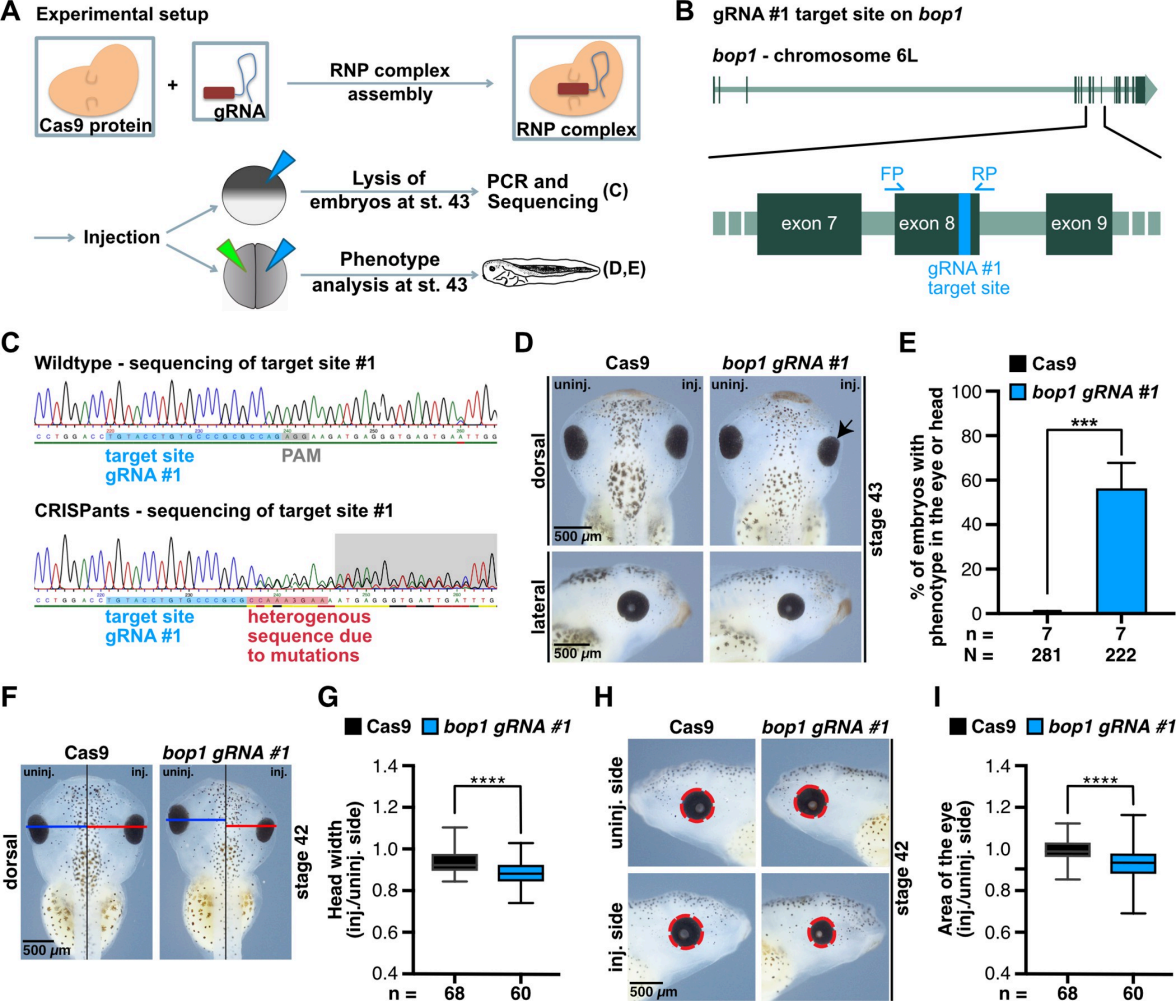
*bop1* knockout using the CIRSPR/Cas9 system. **A** Experimental setup of CRISPR/Cas9 gene editing experiments. Cas9 protein was pre-assembled with *bop1 gRNA #1* to build ribonucleoprotein (RNP) complex. Embryos were injected at one-cell stage for sequencing analysis. Embryos were injected at two-cell stage for phenotypic analysis. **B** Target site of gRNA #1 on *bop1* exon 8. Here shown for chromosome 6L –off note, there are no mismatches between target site on homeologue L and S. **C** DNA pools of 10 wildtype embryos or 10 *bop1* CRISPants were sequenced. Target site of *bop1 gRNA #1* is highlighted in blue, protospacer adjacent motif (PAM) sequence in grey, and heterogenous sequence in CRISPants DNA pool in red. CRISPR/Cas9 system successfully introduced mutations. **D** Injection of *bop1 gRNA #1* resulted in smaller eyes (indicated with black arrow) and smaller heads on the injected side, whereas injection of Cas9 protein alone did not affect anterior development. **E** Statistical analysis of data shown in D. **F** The head width of the injected side (red line) was compared to the width of the uninjected side (blue line). Black line indicates the midline of the embryo. **G** Knockout of *bop1* led to significantly narrower heads on the injected side. **H** Area of the eye on the injected side was compared to the area of the eye on the uninjected side. Measured area is indicated by red dotted line. **I** *bop1* CRISPants showed a significantly reduced eye area. Cas9 protein alone did not affect the area of the eye. Abbreviations: *bop1 gRNA #1*, block of proliferation 1 guide RNA #1; Cas9, CRISPR-associated protein 9; CRISPR, clustered regularly interspaced short palindromic repeats; inj., injected side; n, number of independent experiments; N, number of injected and analyzed embryos; PAM, protospacer adjacent motif; PCR, polymerase chain reaction; RNP, ribonucleoprotein; uninj., uninjected side. Error bars indicate standard error of the means; Whiskers in G and H indicate minimum and maximum. ***, p ≤ 0.001; ****, p ≤ 0.0001.

In addition to the knockdown using MOs, the CRISPR/Cas9 system was used to induce a knockout of *bop1*. Therefore, the synthesized *bop1 gRNA #1* was pre-assembled with the Cas9 protein to form the ribonucleoprotein complex (Fig 5A). The complex was either injected at one-cell stage for further sequencing analysis or at two-cell stage for phenotypic evaluation (Fig 5A).

*bop1 gRNA #1* targets exon 8 of the *bop1* gene (Fig 5B). The wildtype sequence pool showed the CRISPR target site which is followed by the protospacer adjacent motif (PAM) sequence. The DNA sequence of *bop1* CRISPants depicted a heterogeneous sequence starting 4 bases upstream of the PAM sequence indicating a successful gene editing (Fig 5C). *bop1* knockout led to smaller eyes and smaller heads on the injected side (Fig 5D and 5E). Measurements of the head width and the eye area confirmed these findings (Fig 5F–5I). In contrast, Cas9 protein alone did not result in eye or head phenotypes (Fig 5D–5I).

### Knockdown of *bop1* alters the expression of eye marker genes

**Fig 6:**
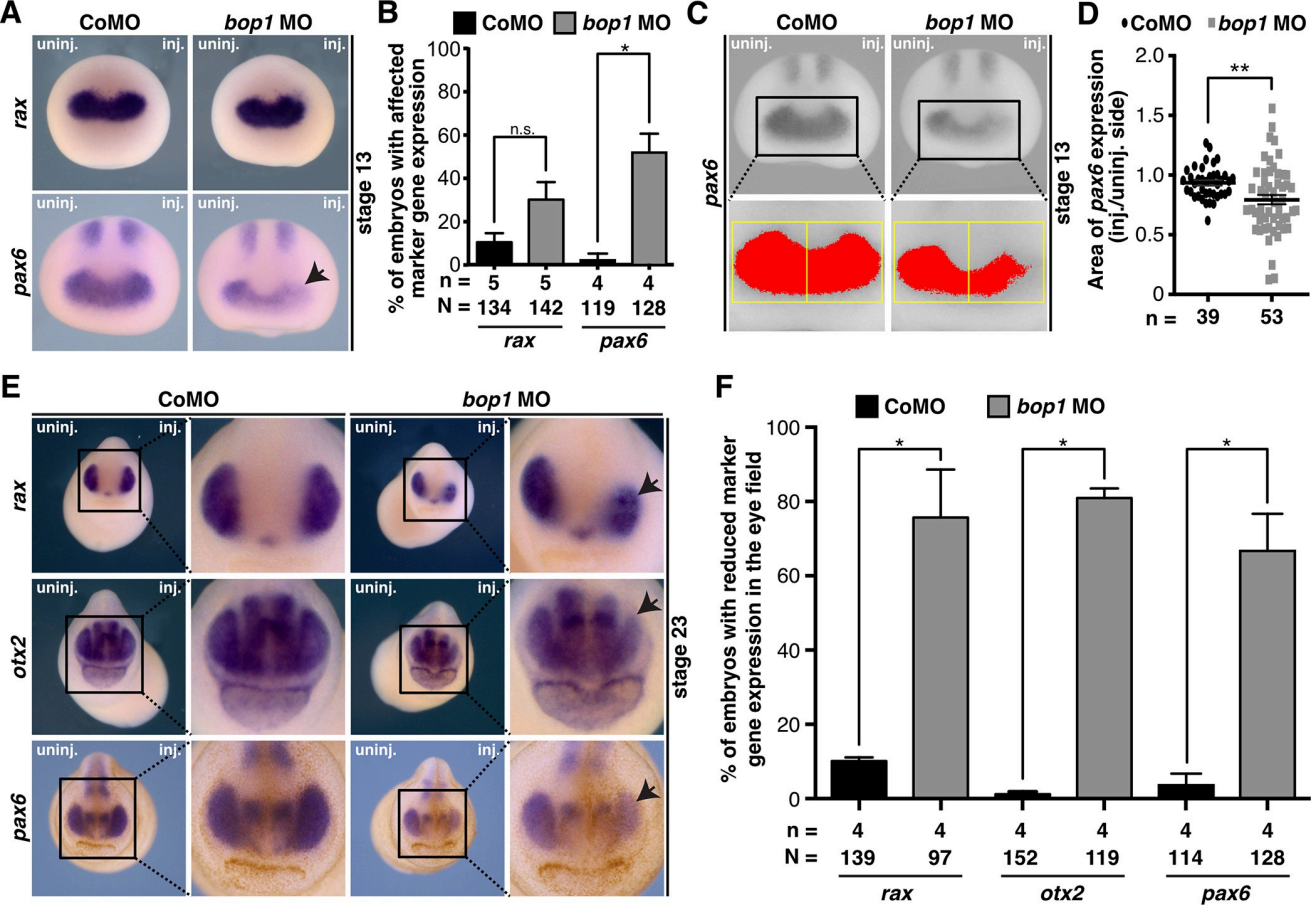
Eye-specific marker genes upon Bop1 deficiency. **A** *Xenopus laevis* embryos at stage 13 injected with 20 ng *bop1* MO or Control MO and analyzed by whole mount *in situ* hybridization. *rax* and *pax6* served as eye-specific marker genes (black arrow indicates location of altered gene expression). Anterior view of embryos is given. **B** Quantitative representation of data given in A. **C** *pax6* expression was analyzed in stage 13 *bop1* morphants (anterior view is shown). Expression domain was photographed and area was measured via ImageJ. Injected side was compared to uninjected side. Yellow boxes indicate area where *pax6* expression (red colored area) was measured. **D** *pax6* expression is significantly reduced on the injected side of *bop1* MO-injected embryos. **E** Stage 23 embryos injected with 20 ng *bop1* MO or Control MO. Anterior view is given. *rax*, *otx2*, and *pax6* were used as eye-specific marker genes (black arrows indicate the location of the altered gene expression). **F** The expression of all three marker genes was significantly reduced in *bop1* morphants. Abbreviations: *bop1* MO, block of proliferation 1 morpholino oligonucleotide; CoMO, Control MO; inj., injected side; n, number of independent experiments; N, number of injected and analyzed embryos; n.s., non-significant; uninj., uninjected side. Error bars indicate standard error of the means; *, p ≤ 0.05; **, p ≤ 0.001.

To analyze the molecular basis of the *bop1* MO-induced eye phenotype, WMISH with well-established eye-specific marker genes was performed. First, we used the eye field-specific marker genes *rax* and *pax6* in stage 13 embryos, when the eye field is induced [50,51]. *rax* expression was not altered upon either *bop1* or Control MO injection (Fig 6A and 6B). *pax6* expression was significantly decreased in the eye field (Fig 6A and 6B), which was also confirmed by expression area measurements (Fig 6C and 6D). At stage 23, *rax*, *otx2*, and *pax6* were used to investigate eye cell differentiation [27]. Here, Bop1 suppression led to a decrease of all three marker genes (Fig 6E and 6F). Control MO-injected embryos showed normally expressed marker genes.

In summary, *bop1* knockdown led to a severe eye phenotype including microphthalmia, coloboma, and disturbed retinal lamination as well as defects in eye-specific marker gene expression during eye field induction and eye cell differentiation.

### Translation, cell proliferation and Tp53 mediated-apoptosis are not affected by *bop1* knockdown

**Fig 7:**
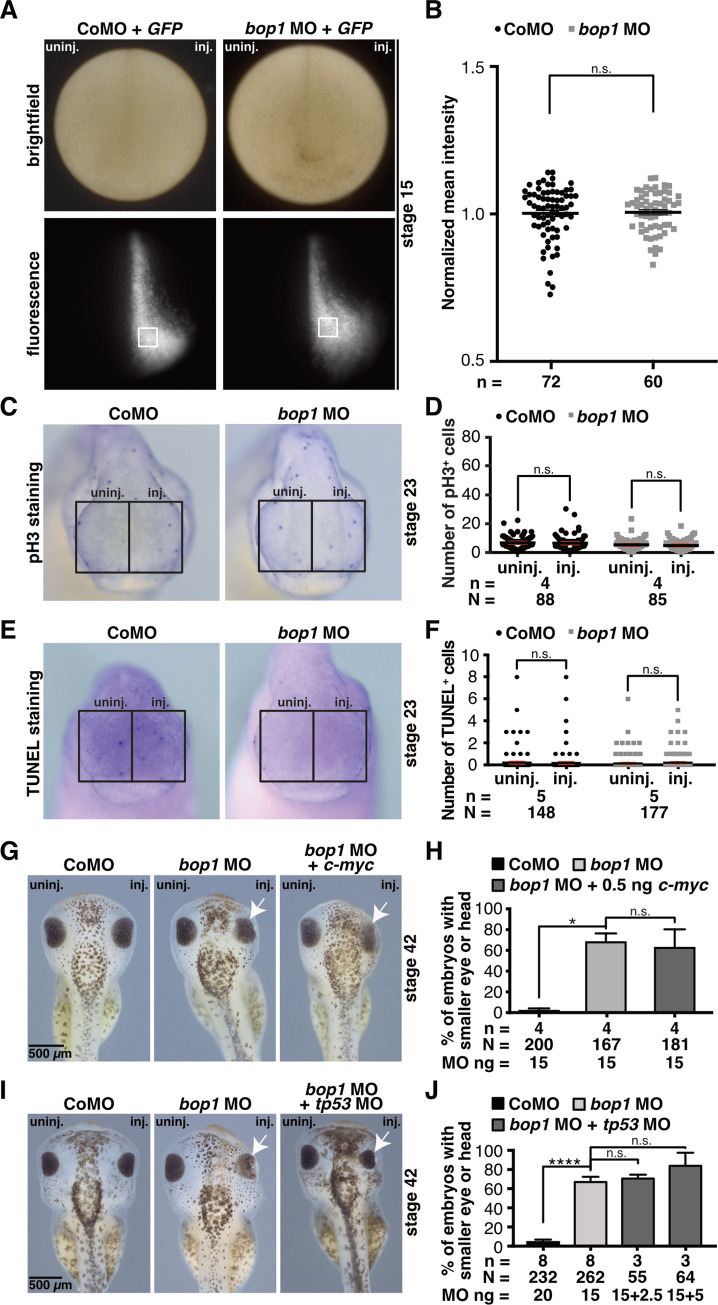
*bop1* knockdown affects neither cell proliferation nor apoptosis. **A** Embryos were injected with Control or *bop1* MO together with 1 ng *GFP* RNA. At stage 15, embryos were photographed using a fluorescence microscope (8-bit greyscale). Intensity of GFP was analyzed by measuring the mean grey value in the indicated areas (white boxes). Mean intensities were normalized to the control group. Anterior view of embryos is given. **B** GFP intensity did not differ between Control MO and *bop1* MO-injected embryos. **C** Anterior view of embryos is shown. Phospho histone 3 (pH3) staining of stage 23 embryos showed no difference upon injection of Control MO or *bop1* MO. **D** Statistical analysis of data given in C. **E** Anterior view of embryos is shown. Terminal deoxynucleotidyl transferase dUTP nick end labeling (TUNEL) staining of stage 23 embryos revealed no difference between Control MO- and *bop1* MO-injected embryos. **F** Statistical evaluation of data given in E. **G** Embryos were injected with Control MO, *bop1* MO, or *bop1* MO + 0.5 ng *c-myc* RNA. At stage 42, embryos were analyzed regarding a phenotype of the anterior tissue. Injected side was compared to uninjected side. White arrows indicate smaller eyes. **H** Statistical analysis of data shown in G reveals that a rescue of the *bop1* MO-induced phenotype is not possible by co-injection of 0.5 ng *c-myc* RNA. **I** Embryos were injected with Control MO, *bop1* MO, or *bop1* MO + *tp53* MO and analyzed at stage 42 regarding a phenotype of the anterior tissue. Co-suppression of Bop1 and Tp53 did not rescue the *bop1* knockdown-associated phenotype. **J** Statistical analysis of data given in I. Abbreviations: *bop1* MO, block of proliferation 1 morpholino oligonucleotide; *c-myc*, *MYC proto-oncogene*; CoMO, Control MO; *GFP*, *green fluorescent protein*; inj., injected side; MO, morpholino oligonucleotide; n, number of independent experiments; N, number of injected embryos and analyzed; n.s., non-significant; pH3, phospho histone 3; tp53, tumor protein p53; TUNEL, terminal deoxynucleotidyl transferase dUTP nick end labeling; uninj., uninjected side. Error bars indicate standard error of the means; *, p ≤ 0.05; ****, p ≤ 0.0001.

Bop1 is a factor necessary for ribosomal biogenesis, therefore we investigated whether *bop1* knockdown possibly affects the translational machinery. Therefore, 1 ng of *GFP* RNA was co-injected with either *bop1* MO or Control MO and GFP intensity was analyzed at stage 15. GFP intensity did not differ between *bop1* morphants and Control MO-injected embryos (Fig 7A and 7B), indicating that—in early *Xenopus* embryos—the translational machinery is not in general affected upon *bop1* knockdown.

Previous studies showed that upon knockdown of ribosomal proteins or factors, proliferative and apoptotic pathways are impaired [17,19,52,53]. Therefore, we investigated the number of proliferative cells via pH3 staining and apoptotic cells using TUNEL staining at stage 23. Neither *bop1* MO nor Control MO injection affected proliferation (Fig 7C and 7D) or apoptosis (Fig 7E and 7F).

In a previous study investigating the ribosomal protein L5 (Rpl5), we have shown that the expression of *c-myc*, which is an important player during ribosomal biogenesis as it enhances the performance of all three polymerases I-III, is reduced in embryos upon *rpl5* knockdown. Furthermore, the co-injection of *c-myc* RNA partially rescued the eye phenotype which occurred upon *rpl5* MO injection [53]. Additionally, Bellmeyer and colleagues showed that *c-myc* knockdown results in a deformed and smaller cranial cartilage similar to the here observed phenotype [54]. Hence, we investigated whether the co-injection of *bop1* MO together with *c-myc* RNA can rescue the *bop1* MO-induced phenotype. The phenotype was not rescued (Fig 7G and 7H).

In defective ribosomal biogenesis, the number of free ribosomal proteins increases. These free proteins bind to MDM2 (Mouse double minute 2 homolog), a crucial regulator of Tp53, thereby stabilizing Tp53 which results in an activation of apoptotic pathways [55]. Furthermore, several studies showed that craniofacial defects can be rescued upon co-knockdown of *Tp53* in mice, frog, and zebrafish [52,56–58]. Therefore, we attempted to rescue the *bop1* MO-induced phenotype at stage 42 by co-injecting *tp53* MO, what failed (Fig 7I and 7J). This finding is in line with the above mentioned TUNEL experiments and indicates that the Bop1 deficiency phenotype is not a result of Tp53 pathway activation.

Conclusively, upon *bop1* knockdown, pathways crucial during defective ribosomal biogenesis, e.g., proliferative and apoptotic pathways, are not impaired. This leads to the hypothesis of Bop1 –additionally to its role in ribosomal biogenesis–implementing a function independent of ribosomal biogenesis during early *Xenopus* development.

### Bop1 functions together with Pax6

We further investigated Pax 6 since 1) Pax6 is a master control gene for eye development [59] and 2) *pax6* expression was strongly reduced upon loss of Bop1 function at the early stage 13 (Figs 3 and 6) when *Xenopus laevis* embryos are independent of *de novo* ribosomal biogenesis due to a maternal supply of ribosomes, proteins, and mRNAs.

**Fig 8:**
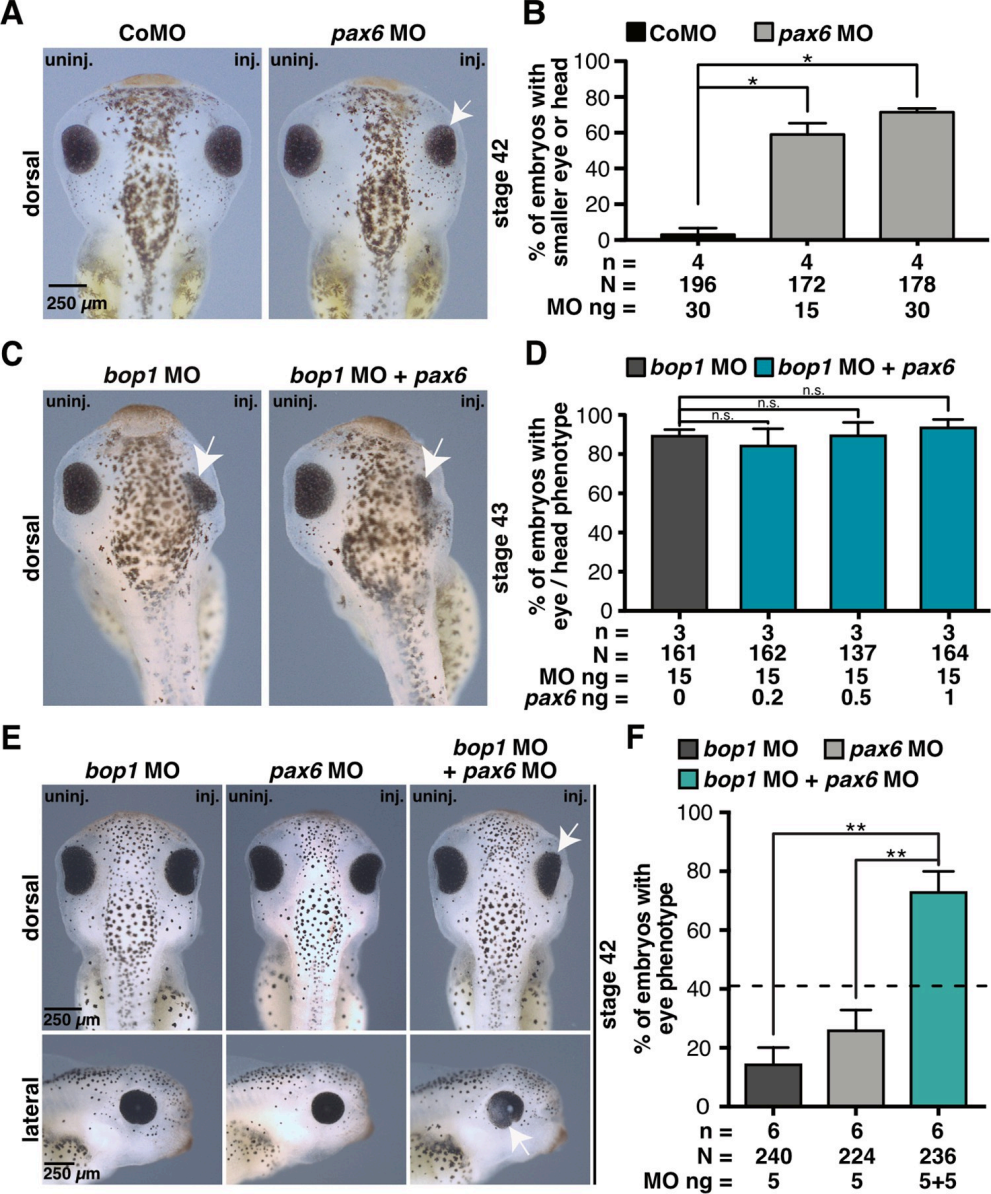
Pax6 and Bop1 act in the same signaling pathway. **A** *Xenopus laevis* embryos were injected with 30 ng Control MO and 15 ng or 30 ng *pax6* MO at eight-cell stage into one animal-dorsal blastomere. At stage 42, embryos were analyzed regarding a phenotype of the anterior tissue. *pax6* MO injection resulted in smaller heads and eyes (white arrows). **B** Statistical evaluation of data given in A. **C** Embryos were injected at eight-cell stage with *bop1* MO alone or in combination with *pax6* RNA. Embryos were fixed at stage 42 and analyzed phenotypically. Smaller eyes are indicated with white arrow. **D** Injection of *bop1* MO alone or in combination with *pax6* RNA resulted in severe eye phenotypes. **E** Injection of 5 ng *bop1* MO or 5 ng *pax6* MO led to a mild eye phenotype in 15% and 27% of the embryos, respectively. The co-injection of both 5 ng *bop1* MO and 5 ng *pax6* MO resulted in a severe eye phenotype in a more than additive manner (73%). **F** Statistical analysis of data given in E. Black dotted line indicates sum of embryos showing an eye or head phenotype injected with 5 ng *bop1* MO and 5 ng *pax6* MO. Abbreviations: *bop1* MO, block of proliferation 1 morpholino oligonucleotide; CoMO, Control MO; inj., injected side; MO, morpholino oligonucleotide; n, number of independent experiments; N, number of injected and analyzed embryos; n.s., non-significant; *pax6* MO, paired box 6 morpholino oligonucleotide; uninj., uninjected side. Error bars indicate standard error of the means; *, p ≤ 0.05; **, p ≤ 0.01.

Previous studies have shown that Pax6 loss of function leads to a severe eye phenotype in *Xenopus* [40] which we confirmed. 15 ng and 30 ng *pax6* MO injected into one animal-dorsal blastomere of eight-cell stage embryos led to smaller eyes and heads in more than 50% of the embryos similar to the *bop1* knockdown phenotype. 30 ng Control MO injection did not reduce eye or head size (Fig 8A and 8B).

To analyze a possible rescue mechanism, *bop1* MO was injected alone and in combination with different amounts of *pax6* RNA. A rescue via *pax6* RNA co-injection was not possible (Fig 8C and 8D). Nevertheless, to characterize a possible common pathway of Bop1 and Pax6, 5 ng *bop1* MO and 5 ng *pax6* MO were injected alone or in combination into one animal-dorsal blastomere of eight-cell stage embryos. The injection of *bop1* MO and *pax6* MO alone led to a mild phenotype in 15% and 26% of the embryos, respectively. The combined injection of both low doses led to a more than additive eye phenotype in 75% of the embryos (Fig 8E and 8F) suggesting a common signaling pathway of the two molecules.

## Discussion

Bop1 is required for processing the large rRNA precursor during ribosome biogenesis. Although *Xenopus* embryos possess a large maternal store of ribosomes, we found a tissue-specific expression of *bop1* during early *Xenopus* embryogenesis. In loss-of-function experiments, proliferation as well as apoptosis were not impaired. However, we found a novel role of this protein during anterior development in that it affects tissue-specific gene expression, especially *pax6*, in a ribosomal independent way.

In our in-depth expression analysis, we provide the first detailed expression pattern of *bop1*. We showed that *bop1* is expressed in neural tissue such as the NCCs, the brain, and the eyes. Of note, *bop1* expression overlaps with the expression of *rax* and *pax6* in the early eye anlage and with *foxd3* at the neural plate border where the NCCs are induced. The here described detailed expression pattern in *Xenopus* is in accordance with earlier findings from Neilson et al. describing *bop1* expression at stage 15 and 28 in *Xenopus tropicalis* [21]. In developing mice, *bop1* expression was also found in the central nervous system, whole brain, and liver [60]. In human fetal tissue, *BOP1* is highly expressed in the brain [61–63]. In conclusion, the conserved expression of *bop1* suggests a role of *bop1* in early neural development.

Here, we showed that knockdown of *bop1* in the anterior tissue of developing *Xenopus laevis* embryos leads to a smaller head width and cranial cartilage structures, a diminished brain size, and malformed and underdeveloped eyes. Our study also found changes in gene expression upon loss of Bop1 and thus provides first clues about the underlying molecular mechanisms. We detected *bop1* expression in *Xenopus* anterior NCCs, and *bop1* knockdown resulted in a broader expression domain for the pan-neural marker gene *sox3*, which was also confirmed using real-time qPCR. Rogers and colleagues already showed that overexpression of *sox3* leads to a severe phenotype in cranial cartilage structures and disturbed formation and migration of NCCs in *Xenopus laevis* embryos [64]. In contrast, expression of *sox2* was not affected upon *bop1* knockdown, showing a specific affect for *sox3*. In our experiments, *bop1* knockdown also resulted in a reduced *foxd3* expression during induction of the neural plate border. Foxd3 has been shown to be required for the epithelial-mesenchymal transition (EMT) of NCCs [65]. Interestingly, BOP1 has been shown to be upregulated in patients with hepatocellular carcinoma as well as gastric cancer and that BOP1 induces EMT, which leads to higher invasiveness and metastasis potential [66,67]. In summary, these data suggest that proper function of Bop1 is required for EMT in general.

The expression of the brain marker genes *otx2*, *en2*, and *egr2* is reduced upon *bop1* knockdown. Besides its role in eye development, Otx2 is also essential for craniofacial development, and mutations in this gene have been associated with brain defects [68,69]. En2 plays an important role in the development of the brain, especially the cerebellum [70], and was found to be associated with infantile autism [71,72]. *En2* knockout mice show dysregulation in monoamine systems and defects in forebrain structures [73]. Expression of *Egr2* is associated with myelination of the peripheral nervous system and the hindbrain region [74], and mutations in the human *EGR2* lead to hereditary myelinopathies [75]. Taken together, the reduction found in marker gene expression in *Xenopus* are in line with the observed NCC and brain phenotypes upon loss of Bop1 function.

Upon *bop1* knockdown, retinal lamination is disturbed and different cell layers are disorganized. In a previous study, we showed that retinal lamination defects can result from inhibited cell adhesion [76], which might also be a possible reason for this phenotype in *bop1* MO-injected embryos. Furthermore, we observed a downregulation of *rax* and *otx2* during eye cell differentiation. Earlier studies by others indicated that mutations in *rax* lead to the problems in eye formation in *Xenopus* and mice [77]. Additionally, previous studies showed that mutations in human *OTX2* and *RAX* lead to anophthalmia, microphthalmia, and coloboma [77–81], findings that are in line with the eye phenotype upon *bop1* knockdown in our study.

Upon *bop1* knockdown using the CRISPR/Cas9 system, *Xenopus laevis* embryos showed eye phenotypes (microphthalmia) and head phenotypes. This is in accordance with our previous results using the antisense-based MO approach.

Our findings are also in line with defects observed upon changes in Bop1 interaction partners. In the developing zebrafish, mutations in *Pes1*, as part of the PeBoW complex, lead to a smaller brain, eye, gut, and liver and a failed expansion of the pancreas [82,83]. In *Xenopus laevis*, the knockdown of both *pes1* and *ppan* (interaction partner of Pes1), also interferes with craniofacial cartilage and eye development [17,19]. The knockdown of the ribosomal biogenesis factor *nucleolar protein 11* leads to a late craniofacial cartilage malformation [52]. Recently, we also have shown that loss of Rpl5 leads to craniofacial cartilage as well as eye defects [53]. In summary, suppression of these investigated ribosomal biogenesis factors (as shown in earlier studies) results in comparable phenotypes as shown for Bop1 in this study, i.e., a disrupted development of the cartilaginous head structures, the brain, and the eye. Interestingly, although the characteristics of these phenotypes differ somewhat from those found in ribosomopathies, they have a few features in common, including eye abnormalities and craniofacial dysmorphology [3,84–90].

Knockdown of *bop1* affects marker gene expression in very early stages of development, i.e., even before exhaustion of the maternal ribosome pool. *Xenopus* embryos possess a pool of around 10^12^ maternal ribosomes, as well as mRNAs and proteins, which together are sufficient for development until approximately stage 26 [16,19]. Therefore, in their first days of development *Xenopus* embryos are not dependent on zygotic ribosome biosynthesis. In line with this finding, we showed in previous work that interfering with the processing of the 45S rRNA precursor does not affect early development of *Xenopus*, in particular during neural and pronephric development [19].

At stage 15, we found GFP expression not to be affected in *bop1* morphants, indicating that the translational machinery is not disturbed in general. This strengthens the hypothesis that Bop1 might have an additional, ribosomal independent function as the knockdown of this ribosomal factor does not affect the translational machinery and *de novo* ribosomal biogenesis is less important during early development due to the maternal store which is sufficient until swimming tadpole stage [12].

Analyzing proliferative and apoptotic processes via pH3 and TUNEL staining, we found that *bop1* knockdown did neither affect proliferation nor apoptosis. This is not in line with the fact that ribosomes are crucial for cell proliferation. In a previous study, we were able to partially rescue an eye phenotype, which occurred upon knockdown of a ribosomal protein, with *c-myc* RNA [53]. Hence, we analyzed whether *c-myc* RNA, as transcriptional enhancer important for proliferation, can rescue the *bop1* MO-induced phenotype. The phenotype was not rescued, again hinting towards a ribosomal independent function of Bop1.

In previous studies, Tp53 was found to be activated during pathological processes of ribosomal biogenesis, e.g., during Diamond-Blackfan anemia [91–93]. Wu and colleagues demonstrated that lower BOP1 levels lead to decreased ribosome biogenesis, which results in Tp53-dependent proliferative inhibition, oxidative stress, and apoptosis in a human cell line [94]. This finding is in contrast with our findings because we found that the *tp53* knockdown did not rescue the *bop1* knockdown-associated phenotype. The divergent results can be explained by the theory that, as explained above, *Xenopus* embryos rely on maternal ribosomes during early development and that zygotic ribosome biogenesis does not start at a relevant rate until the mid-20 stages at the earliest [16]. Accordingly, as mentioned in the introduction, for the ribosomal factor Ppan we were able to show a Tp53-independent nucleolar stress response pathway [20]. In conclusion, we propose that the early defects observed in *Xenopus* in our study can probably not be explained by defective ribosomal biogenesis. Therefore, understanding the phenotype upon *bop1* knockdown in *Xenopus* might provide novel insights into the pathogenesis of ribosomopathies.

Our findings of altered gene expression provide an indication of the underlying mechanism. We found that the expression of *pax6* was reduced upon *bop1* knockdown. Previous studies demonstrated that downregulation of Pax6 leads to smaller or absent eyes in *Xenopus* and mice [40,95,96], which is in line with our result that *pax6* knockdown in anterior tissue leads to a similar phenotype as *bop1* knockdown. A rescue using *pax6* RNA was not possible. This might be due to the reason that other molecules and pathways, e.g., *sox3-* or *foxd3-*related pathways are affected upon *bop1* knockdown as well. Nevertheless, by co-injecting *bop1* MO and *pax6* MO, we showed a synergistic relationship between Bop1 and Pax6. These results strengthen the hypothesis that Bop1 and Pax6 act in the same signaling pathway. Previous studies found that in mice and *Xenopus*, the overexpression of *pax6* leads to several eye defects, including microphthalmia, ectopic eyes, and anophthalmia [97,98]. Also, mutations in human *PAX6* lead to numerous eye defects, including aniridia, corneal opacification, and cataract [99]. In conclusion, our results alongside with previous studies by others, indicate that Pax6 expression needs to be finely balanced to ensure proper eye development and that the knockdown of *bop1* and the resulting changes in *pax6* expression might contribute to the *bop1* MO-induced phenotype.

## Conclusion and outlook

Taken together, we showed that Bop1 is crucial for *Xenopus* anterior development. The results demonstrated that loss of Bop1 phenotypes closely resembles the clinical manifestations of ribosomopathies. However, the early effect on embryonic development suggests an extra-ribosomal function of Bop1 via a Pax6-mediated mechanism.

Because Bop1 is a member of the PeBoW complex and the stability of each protein is interdependent, it would be of high interest to analyze the expression and behavior of the two complex partners Pes1 and WDR12 upon *bop1* knockdown. How Bop1 regulates gene expression on a molecular level awaits further elucidation.

## Supporting information

S1 Fig Synteny analysis and comparison of block of proliferation 1 protein domains between different species. **A** Synteny analysis of *bop1* in *Homo sapiens*, *Mus musculus*, *Xenopus laevis L*, *Xenopus laevis S*, and *Danio rerio*. The genomic region next to *bop1* is conserved across the different species. **B, C** Protein domains of block of proliferation 1 (Bop1). The BOP1 N-terminal (NT) domain is depicted in orange and the WD 40 repeat (WD 40) domain, in beige. **D** The protein length (number of amino acids), the overall homology (%), the Bop1 NT domain homology (%) and the WD40 domain homology (%) of *Homo sapiens*, *Mus musculus*, *Xenopus laevis L*, *Xenopus laevis S*, and *Danio rerio* were compared. Bop1 is highly conserved across species. Abbreviations: aa, amino acid; Bop1, block of proliferation 1.
(TIFF)

S2 Fig MO specificity and effectiveness test. **A** Binding sites of *bop1* MO (blue) and *bop1* MO2 (turquoise) are highlighted in the *Xenopus bop1* gene (L and S- form). The start codon is indicated in orange. **B** Binding sites of *bop1* MO (on both homeologues), *bop1* MO2 (on both homeologues), and the Δ*5’UTR-bop1* construct on *Xenopus bop1*. Start codon is indicated in orange. Blue letters indicate differences between binding sites of chromosome S and chromosome L. Red letters indicate differences between binding sites of *bop1* MO and Δ*5’UTR-bop1*. **C** Binding specificity test of *bop1* MO. Injection of 10 ng Control MO along with 1 ng *bop1 MO bs-GFP* or 1 ng *bop1 MO bs chr*. *L-GFP* led to *GFP* translation. Co-injection of 10 ng *bop1* MO along with 1 ng *bop1 MO bs-GFP* or with 1 ng *bop1 MO bs chr*. *L-GFP* efficiently blocked *GFP* expression. However, co-injection of Δ*5’UTR-bop1 MO bs-GFP* with *bop1* MO led to *GFP* translation. **D** Binding specificity test of *bop1* MO2. Injection of 10 ng Control MO together with 1 ng *bop1 MO2 bs-GFP* or with 1 ng *bop1 MO2 bs chr*. *L-GFP* resulted in *GFP* translation. In contrast, injection of *bop1* MO2 together with either 1 ng *bop1 MO2 bs-GFP* or 1 ng *bop1 MO2 bs chr*. *L-GFP* blocked *GFP* translation. Numbers below fluorescence photos describe number of fluorescent embryos. Abbreviations: *bop1* MO, block of proliferation 1 morpholino oligonucleotide; bs, binding site; CoMO, Control morpholino oligonucleotide; GFP, green fluorescent protein; UTR, untranslated region.
(TIFF)

S3 Fig Eye and head phenotype upon *bop1* MO2 injection. **A** Comparison of the head width of injected (red line) to un-injected (blue line) sides after *bop1* MO2, Control MO, or *bop1* MO2 together with 0.5 ng of Δ*5’UTR-bop1* RNA injection at stage 42. **B** Statistical evaluation of data in A. The head size of the *bop1* MO2-injected side was significantly reduced compared to the Control MO-injected and un-injected side. Co-injection of 0.5 ng of Δ*5’UTR-bop1* RNA rescued the head phenotype. **C** Knockdown of *bop1* by *bop1* MO2 led to a severe eye phenotype, with underdeveloped or malformed eyes (black arrows) in stage 42 embryos. This eye phenotype was rescued upon co-injection of 0.5 ng Δ*5’UTR-bop1* RNA. **D** Statistical evaluation of data in C. *bop1* MO2, *bop1* MO2 + *Δ5’UTR-bop1* RNA and Control MO-injected side was compared to un-injected side of embryos. **E** The area of the eye was measured (red dotted circle). **F** Statistical analysis showed significantly smaller eyes in embryos injected with *bop1* MO2. The phenotype was rescued in embryos injected with *bop1* MO2 together with 0.5 ng Δ*5’UTR-bop1* RNA. **G** The angle of eye fissure (red angle) was measured and *bop1* MO2, *bop1* MO2 + *Δ5’UTR-bop1* RNA and Control MO-injected embryos were compared to the uninjected side. **H** Embryos developed colobomas upon *bop1* MO2 injection, whereas co-injection of 0.5 ng Δ*5’UTR-bop1* RNA rescued this coloboma phenotype. Abbreviations: *bop1* MO2, block of proliferation 1 morpholino oligonucleotide 2; CoMO, Control MO; inj., injected side; MO, morpholino oligonucleotide; n, number of independent experiments; N, number of injected and analyzed embryos; uninj., un-injected side. *bop1* is the Δ*5’UTR-bop1* RNA used for rescues. Error bars indicate standard error of the means; Whiskers in D indicate minimum and maximum. **, p <0.01; ***, p < 0.001; ****, p < 0.0001.
(TIFF)

S4 Fig Overexpressing *bop1* does not affect anterior development in *Xenopus laevis*. *bop1* knockdown-associated phenotype is not rescued by co-injecting *foxd3* RNA. **A** Overexpression of *bop1* did not result in a phenotype of anterior neural tissue in stage 42 embryos. *bop1* RNA and *GFP* RNA-injected side was compared to un-injected side of embryos. **B** Statistical evaluation of data given in A. **C** Co-injection of *bop1* MO and *foxd3* RNA did not rescue the cranial cartilage phenotype. *bop1* MO, Control MO, and *bop1* MO + *foxd3* RNA-injected side was compared to un-injected side of stage 45 embryos. Black arrows indicate a smaller cranial cartilage. **D** Statistical evaluation of data in C. **E** Alcian blue stained cranial cartilages of stage 45 embryos showed a reduced cartilage upon *bop1* MO and *bop1* MO + *foxd3* RNA injection. Branchial arches (ba), Meckel´s cartilage (mc), tectum anterius (ta) were mostly affected (black arrows). Abbreviations: ba, branchial arches; *bop1* MO, block of proliferation 1 morpholino oligonucleotide; CoMO, Control MO; *GFP*, *green fluorescent protein*; inj., injected side; mc, Meckel´s cartilage; MO, morpholino oligonucleotide; n, number of independent experiments; N, number of injected and analyzed embryos; n.s., non-significant; ta, tectum anterius; uninj., un-injected side. Error bars indicate standard error of the means; ****, p<0.0001.
(TIFF)
