# Supplementary figures and images for: Functions of block of proliferation 1 during anterior development in Xenopus laevis
Source: PLoS One. 2022 Aug 25;17(8):e0273507. doi: 10.1371/journal.pone.0273507 (PMC9409556; doi:10.1371/journal.pone.0273507)

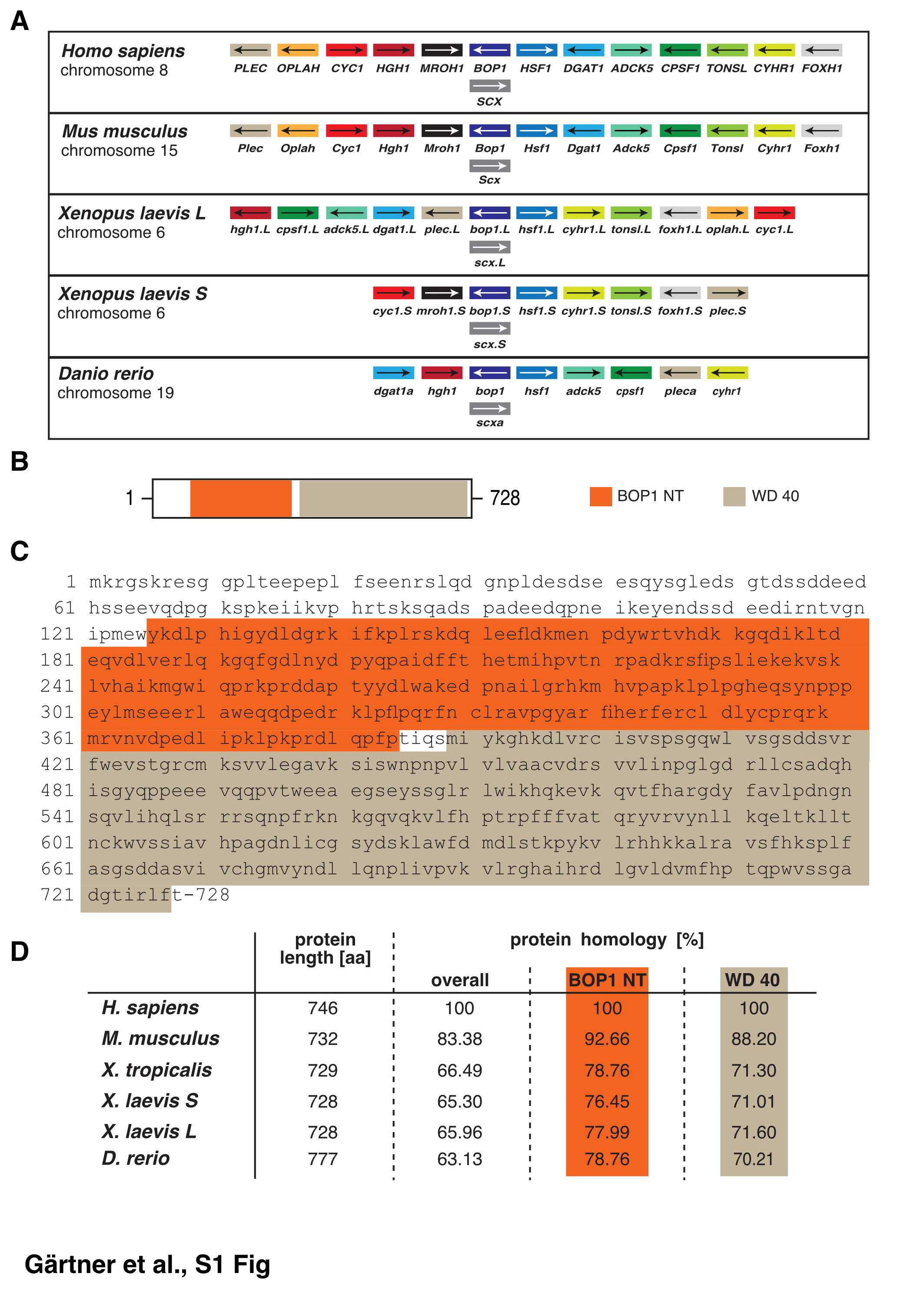

Supplement: S1 Fig — A Synteny analysis of bop1 in Homo sapiens, Mus musculus, Xenopus laevis L, Xenopus laevis S, and Danio rerio. The genomic region next to bop1 is conserved across the different species. B, C Protein domains of block of proliferation 1 (Bop1). The BOP1 N-terminal (NT) domain is depicted in orange and the WD 40 repeat (WD 40) domain, in beige. D The protein length (number of amino acids), the overall homology (%), the Bop1 NT domain homology (%) and the WD40 domain homology (%) of Homo sapiens, Mus musculus, Xenopus laevis L, Xenopus laevis S, and Danio rerio were compared. Bop1 is highly conserved across species. Abbreviations: aa, amino acid; Bop1, block of proliferation 1. (TIFF) [file pone.0273507.s001.tiff]

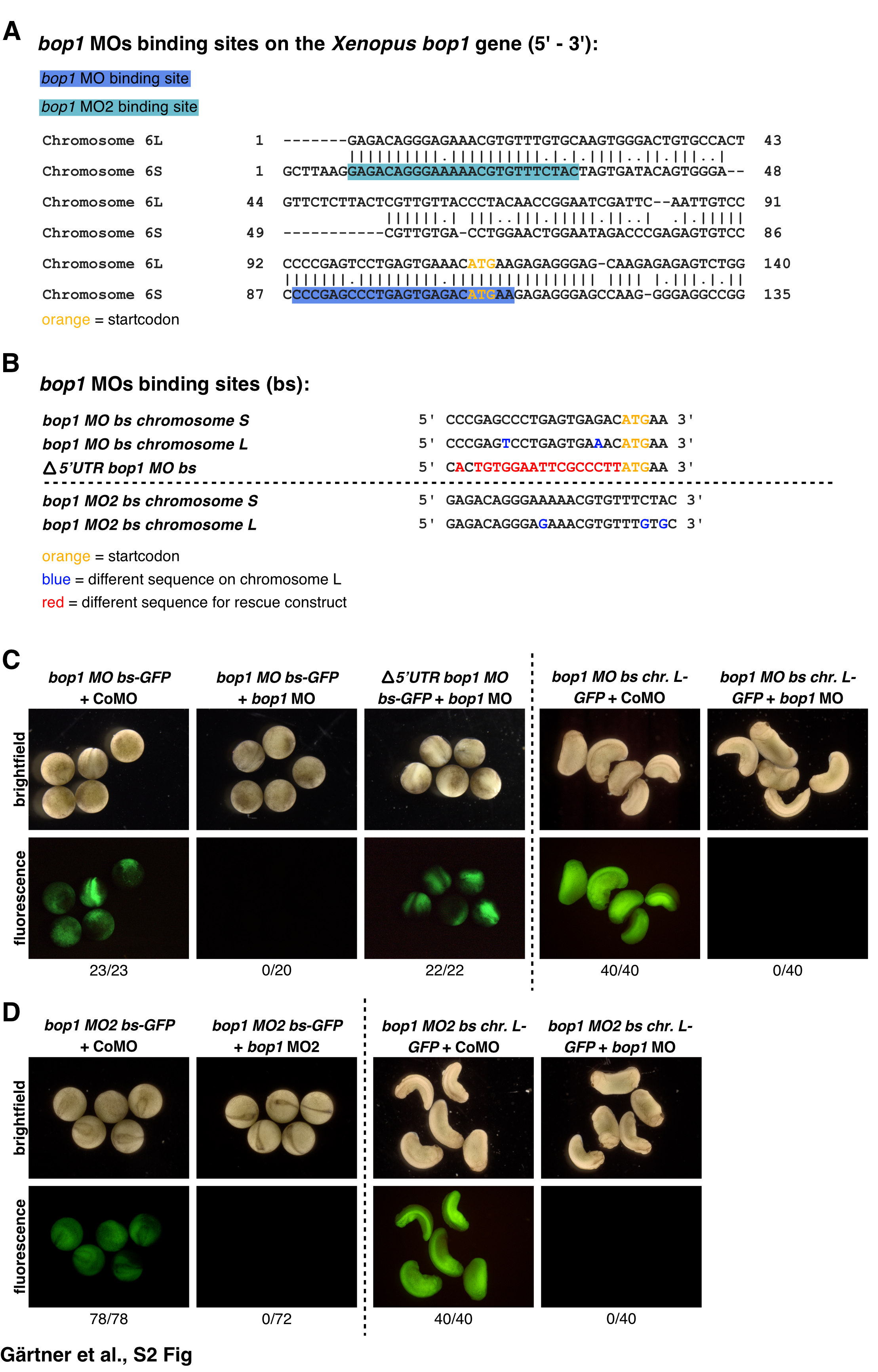

Supplement: S2 Fig — A Binding sites of bop1 MO (blue) and bop1 MO2 (turquoise) are highlighted in the Xenopus bop1 gene (L and S- form). The start codon is indicated in orange. B Binding sites of bop1 MO (on both homeologues), bop1 MO2 (on both homeologues), and the Δ5’UTR-bop1 construct on Xenopus bop1. Start codon is indicated in orange. Blue letters indicate differences between binding sites of chromosome S and chromosome L. Red letters indicate differences between binding sites of bop1 MO and Δ5’UTR-bop1. C Binding specificity test of bop1 MO. Injection of 10 ng Control MO along with 1 ng bop1 MO bs-GFP or 1 ng bop1 MO bs chr. L-GFP led to GFP translation. Co-injection of 10 ng bop1 MO along with 1 ng bop1 MO bs-GFP or with 1 ng bop1 MO bs chr. L-GFP efficiently blocked GFP expression. However, co-injection of Δ5’UTR-bop1 MO bs-GFP with bop1 MO led to GFP translation. D Binding specificity test of bop1 MO2. Injection of 10 ng Control MO together with 1 ng bop1 MO2 bs-GFP or with 1 ng bop1 MO2 bs chr. L-GFP resulted in GFP translation. In contrast, injection of bop1 MO2 together with either 1 ng bop1 MO2 bs-GFP or 1 ng bop1 MO2 bs chr. L-GFP blocked GFP translation. Numbers below fluorescence photos describe number of fluorescent embryos. Abbreviations: bop1 MO, block of proliferation 1 morpholino oligonucleotide; bs, binding site; CoMO, Control morpholino oligonucleotide; GFP, green fluorescent protein; UTR, untranslated region. (TIFF) [file pone.0273507.s002.tiff]

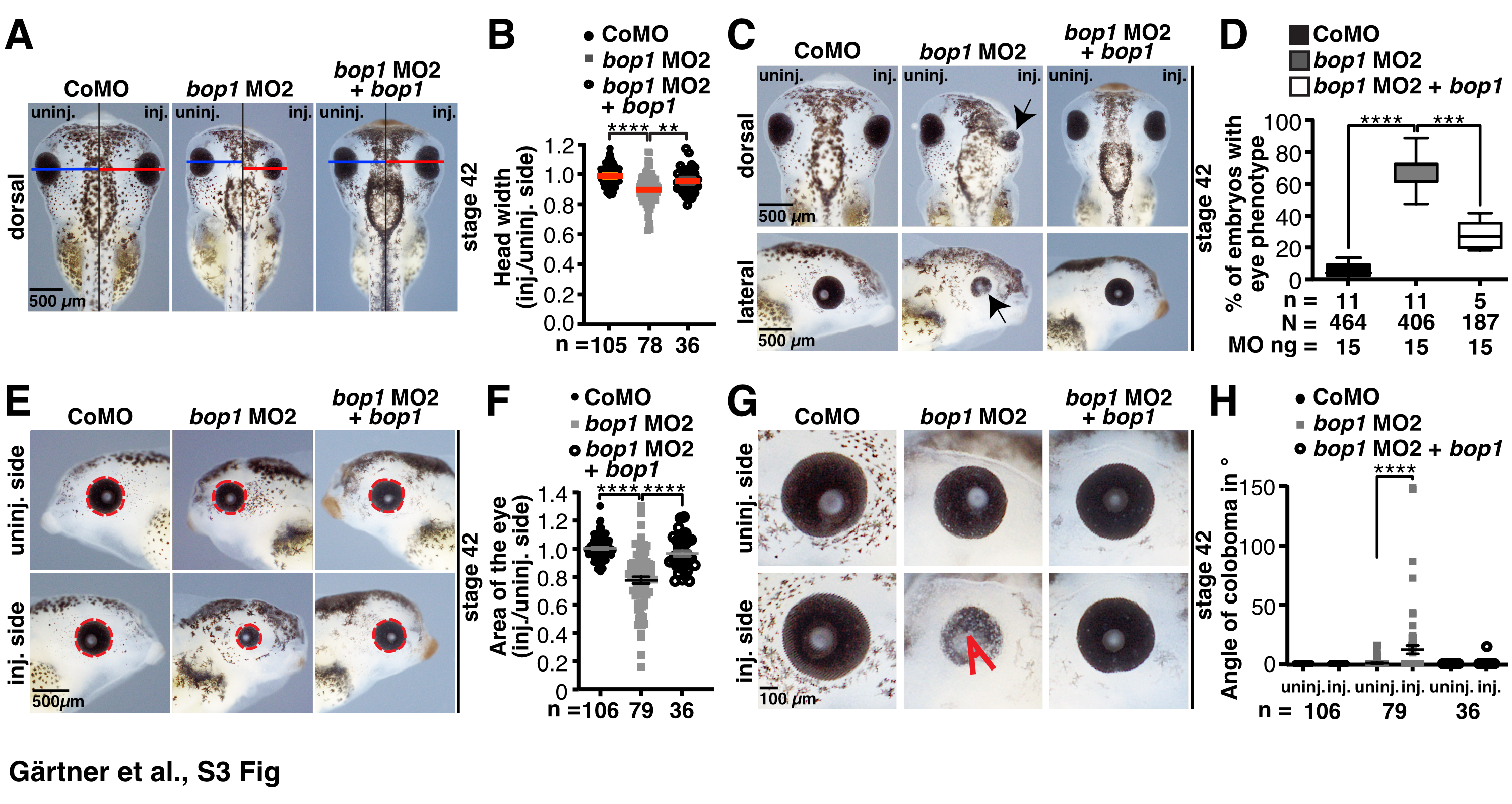

Supplement: S3 Fig — A Comparison of the head width of injected (red line) to un-injected (blue line) sides after bop1 MO2, Control MO, or bop1 MO2 together with 0.5 ng of Δ5’UTR-bop1 RNA injection at stage 42. B Statistical evaluation of data in A. The head size of the bop1 MO2-injected side was significantly reduced compared to the Control MO-injected and un-injected side. Co-injection of 0.5 ng of Δ5’UTR-bop1 RNA rescued the head phenotype. C Knockdown of bop1 by bop1 MO2 led to a severe eye phenotype, with underdeveloped or malformed eyes (black arrows) in stage 42 embryos. This eye phenotype was rescued upon co-injection of 0.5 ng Δ5’UTR-bop1 RNA. D Statistical evaluation of data in C. bop1 MO2, bop1 MO2 + Δ5’UTR-bop1 RNA and Control MO-injected side was compared to un-injected side of embryos. E The area of the eye was measured (red dotted circle). F Statistical analysis showed significantly smaller eyes in embryos injected with bop1 MO2. The phenotype was rescued in embryos injected with bop1 MO2 together with 0.5 ng Δ5’UTR-bop1 RNA. G The angle of eye fissure (red angle) was measured and bop1 MO2, bop1 MO2 + Δ5’UTR-bop1 RNA and Control MO-injected embryos were compared to the uninjected side. H Embryos developed colobomas upon bop1 MO2 injection, whereas co-injection of 0.5 ng Δ5’UTR-bop1 RNA rescued this coloboma phenotype. Abbreviations: bop1 MO2, block of proliferation 1 morpholino oligonucleotide 2; CoMO, Control MO; inj., injected side; MO, morpholino oligonucleotide; n, number of independent experiments; N, number of injected and analyzed embryos; uninj., un-injected side. bop1 is the Δ5’UTR-bop1 RNA used for rescues. Error bars indicate standard error of the means; Whiskers in D indicate minimum and maximum. **, p <0.01; ***, p < 0.001; ****, p < 0.0001. (TIFF) [file pone.0273507.s003.tiff]

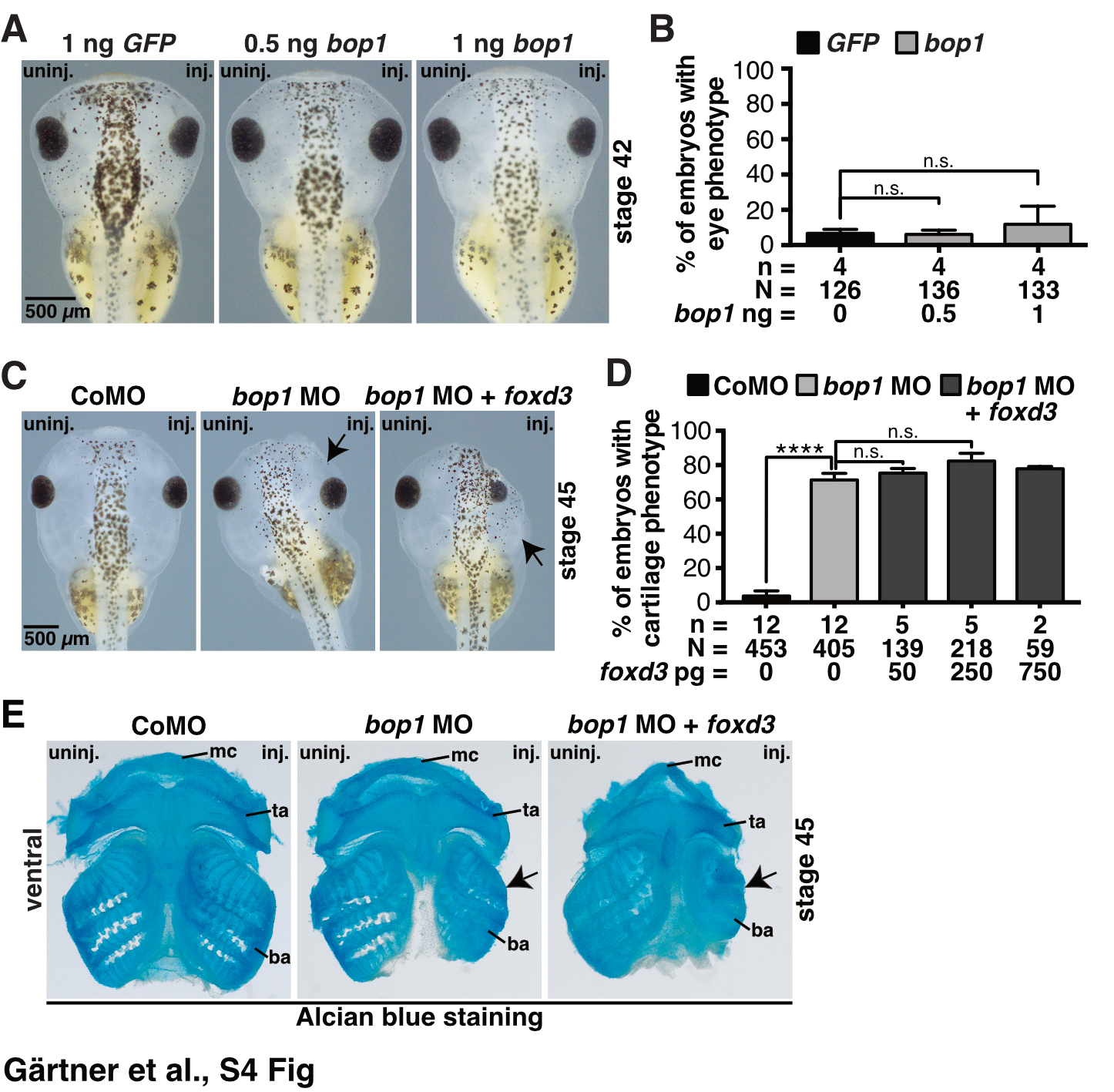

Supplement: S4 Fig — A Overexpression of bop1 did not result in a phenotype of anterior neural tissue in stage 42 embryos. bop1 RNA and GFP RNA-injected side was compared to un-injected side of embryos. B Statistical evaluation of data given in A. C Co-injection of bop1 MO and foxd3 RNA did not rescue the cranial cartilage phenotype. bop1 MO, Control MO, and bop1 MO + foxd3 RNA-injected side was compared to un-injected side of stage 45 embryos. Black arrows indicate a smaller cranial cartilage. D Statistical evaluation of data in C. E Alcian blue stained cranial cartilages of stage 45 embryos showed a reduced cartilage upon bop1 MO and bop1 MO + foxd3 RNA injection. Branchial arches (ba), Meckel´s cartilage (mc), tectum anterius (ta) were mostly affected (black arrows). Abbreviations: ba, branchial arches; bop1 MO, block of proliferation 1 morpholino oligonucleotide; CoMO, Control MO; GFP, green fluorescent protein; inj., injected side; mc, Meckel´s cartilage; MO, morpholino oligonucleotide; n, number of independent experiments; N, number of injected and analyzed embryos; n.s., non-significant; ta, tectum anterius; uninj., un-injected side. Error bars indicate standard error of the means; ****, p<0.0001. (TIFF) [file pone.0273507.s004.tiff]
